# Weak tension accelerates hybridization and dehybridization of short oligonucleotides

**DOI:** 10.1093/nar/gkad118

**Published:** 2023-03-03

**Authors:** Derek J Hart, Jiyoun Jeong, James C Gumbart, Harold D Kim

**Affiliations:** School of Physics, Georgia Institute of Technology, 837 State Street, Atlanta, GA 30332-0430, USA; School of Physics, Georgia Institute of Technology, 837 State Street, Atlanta, GA 30332-0430, USA; School of Physics, Georgia Institute of Technology, 837 State Street, Atlanta, GA 30332-0430, USA; School of Physics, Georgia Institute of Technology, 837 State Street, Atlanta, GA 30332-0430, USA

## Abstract

The hybridization and dehybridization of DNA subject to tension is relevant to fundamental genetic processes and to the design of DNA-based mechanobiology assays. While strong tension accelerates DNA melting and decelerates DNA annealing, the effects of tension weaker than 5 pN are less clear. In this study, we developed a DNA bow assay, which uses the bending rigidity of double-stranded DNA (dsDNA) to exert weak tension on a single-stranded DNA (ssDNA) target in the range of 2–6 pN. Combining this assay with single-molecule FRET, we measured the hybridization and dehybridization kinetics between a 15 nt ssDNA under tension and a 8–9  nt oligonucleotide, and found that both the hybridization and dehybridization rates monotonically increase with tension for various nucleotide sequences tested. These findings suggest that the nucleated duplex in its transition state is more extended than the pure dsDNA or ssDNA counterpart. Based on coarse-grained oxDNA simulations, we propose that this increased extension of the transition state is due to steric repulsion between the unpaired ssDNA segments in close proximity to one another. Using linear force-extension relations verified by simulations of short DNA segments, we derived analytical equations for force-to-rate conversion that are in good agreement with our measurements.

## INTRODUCTION

DNA strand separation or unzipping followed by annealing or rezipping is commonplace in many fundamental genomic processes such as homologous recombination and R-loop formation (1–6). Although genomic processes inside the cell are orchestrated by motor proteins or enzymes, they are thought to be aided by intrinsic dynamics of the underlying genomic DNA (7–11). Therefore, thermally-induced separation of duplex DNA into single strands and its reverse reaction may play an important role in active genomic processes. For example, in both prokaryotic and eukaryotic genomes, origins of replication commonly feature a 10–100 bp DNA unwinding element, whose weak duplex stability determines origin function (12–14). In CRISPR-Cas systems, melting is a rate-limiting step for Cas9 target selection, and has also been found to induce off-target binding and cleavage (15–17).

The melting probability of a duplex region depends not only on its sequence (18), but also on the local stress (18–22). The genomic DNA *in vivo* is seldom in a relaxed state, but rather is subjected to various forms of stress: bending, twisting, and tension. Several DNA force spectroscopy experiments have carefully explored how melting is affected by a strong artificial tension (23–26), but the effect of weak tension (<5 pN), which is arguably more relevant to genomic processes *in vivo* or DNA-based systems *in vitro*, is less clear. Forces in this range can be exerted on a duplex region during active processes such as loop extrusion by SMC complexes (27,28) and also by thermal fluctuations of flanking DNA segments (29). Molecules involved in cell mechanotransduction also regularly experience forces at this scale (30). Therefore, understanding the effect of weak tension on DNA hybridization/dehybridization can elucidate the physical regulation of genomic processes, and aid our design of DNA-based force sensors and actuators for the study of cell signaling mechanics (31–36) and the control of DNA nanostructures (37,38).

**Figure 1.**
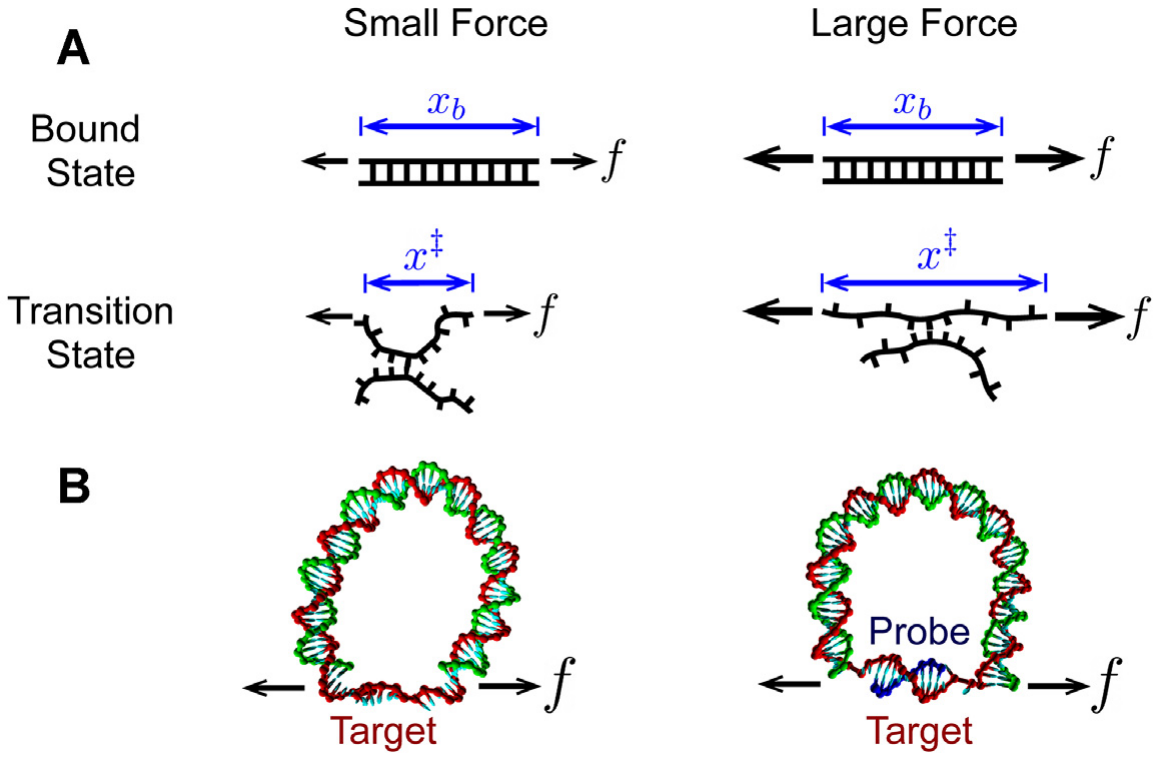
Force-dependent DNA extension. (**A**) A proposed model for how the extension of a duplex differs between small and large forces. The elasticity of the bound state is rigid, and therefore its extension *x*_*b*_ is mostly unaffected by force. On the other hand, the transition state may be more flexible, in which case its extension *x*^‡^ will be force-dependent. In this case, worm-like chain models predict that at large forces, *x*^‡^ > *x*_b_, whereas at small forces *x*^‡^ < *x*_b_. (**B**) Force generation with a DNA bow. A bent bow-like duplex of variable length exerts tension on a 15 nt ssDNA target (bowstring), extending the strand.

In general, the force (*f*) dependence of two-state binding and unbinding kinetics can be modeled with a one-dimensional extension coordinate *x* as (39,40)


(1) $$\begin{equation*} k_\alpha (f)=k_\alpha (0) \exp \left(\int _0^f\Delta x^\ddagger (f^\prime )df^\prime /k_\mathrm{B}T\right), \end{equation*}$$

where *k*_α_ is the rate constant for binding (α = on) or unbinding (α = off), Δ*x*^‡^ is the extension of the transition state (*x*^‡^) relative to the unbound (*x*_u_) or bound state (*x*_b_), and *k*_B_*T* is the thermal energy. If the transition state is more extended than the bound state by a constant (Δ*x*^‡^ > 0), Equation (1) yields the well-known Bell’s formula (41): *k*_off_ ∼ exp (*f*Δ*x*^‡^/*k*_B_*T*), which predicts that *k*_off_ monotonically increases with force. For DNA hybridization/dehybridization, the transition state is thought to be a nucleated duplex that contains both single-stranded DNA (ssDNA) and double-stranded DNA (dsDNA) (42–44). According to the worm-like chain model, ssDNA, whose persistence length (*P*) is ∼1 nm, behaves like a flexible chain in the low force regime (*f* < *k*_B_*T*/*P* ∼ 5 pN) (45). It is thus conceivable that the transition state could be less extended than the pure dsDNA state in the low force regime (Figure 1A and Supplementary Figure S1). Based on this idea, it was recently proposed that *k*_off_(*f*) can decrease with force until *f* ∼ 5 pN before increasing in the high force regime (40,46,47). This counter-intuitive effect known as ‘roll-over’ was predicted in a recent single-molecule fluorescence-tweezers experiment (48), but the limited data leave the conclusion in question. Furthermore, how the extension of the nucleated duplex in the transition state compares to that of dsDNA in the bound state and pure ssDNA in the unbound state is not known.

**Figure 2.**
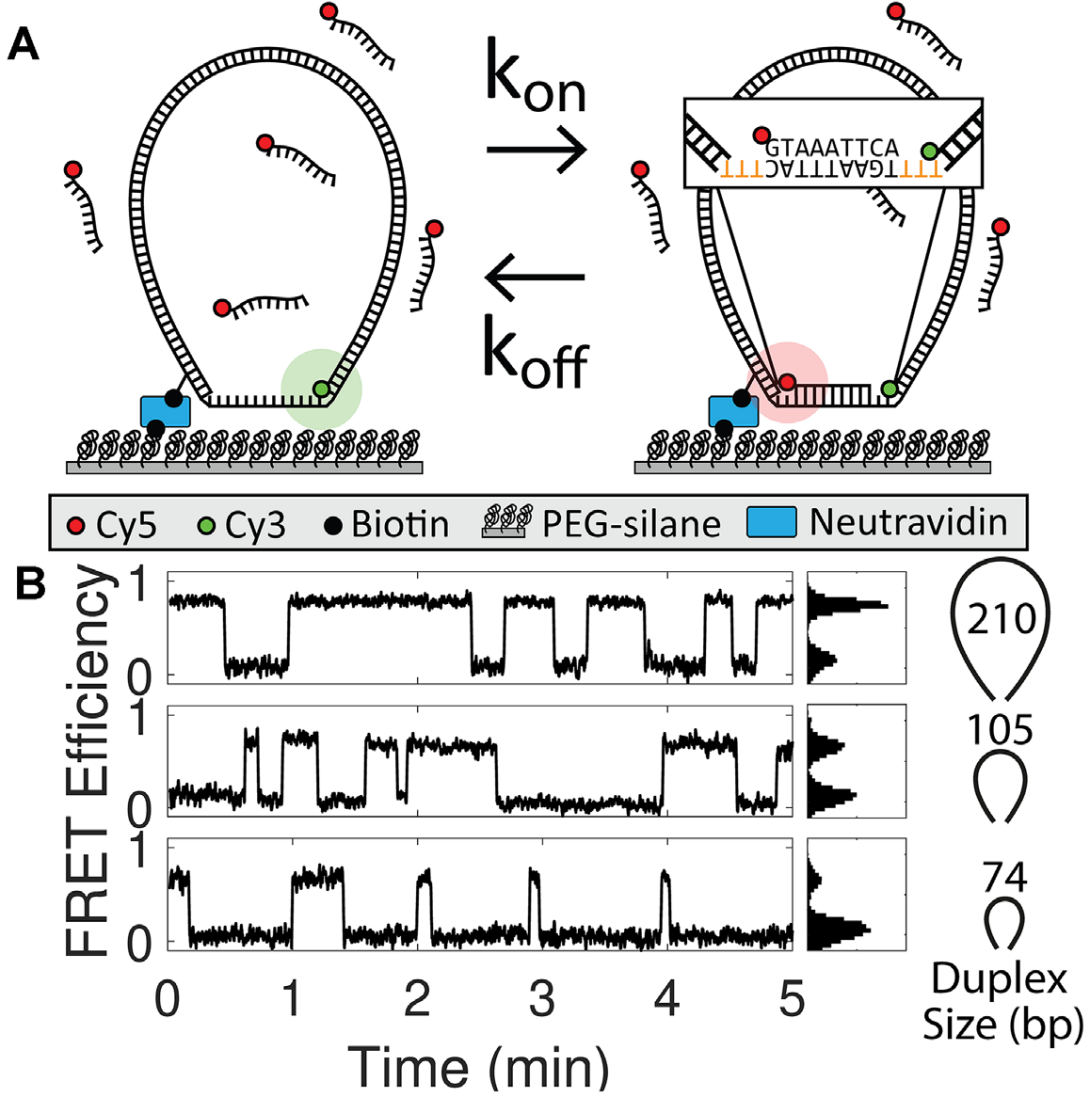
FRET-based DNA bow assay. (**A**) Schematic of DNA bow assay FRET setup. Cy3-labeled DNA bows are immobilized on a PEGylated coverslip and excited by an evanescent wave of a 532-nm laser using TIRF microscopy. The inset highlights the ssDNA sequence (TGAAATTAC) targeted by the Cy5-labeled probe (GTAAATTCA). To avoid additional stacking interactions between the probe and the DNA bow, the 9 nt target segment was flanked by 3 nt ssDNA gaps (highlighted orange) in all construct designs. (**B**) Example FRET efficiency traces for three different dsDNA arc lengths (210, 105, 74 bp) exerting three separate forces (1.8, 3.8, 6.3 pN, respectively). FRET histograms are shown right. Binding and unbinding rates are extracted from the mean dwell times of low and high FRET states respectively.

Here, we developed a DNA construct dubbed ‘DNA bow’ (Figure 1B) to exert tension in the range between 2 and 6 pN on a short DNA oligonucleotide. The DNA bow is composed of a dsDNA segment (arc) of variable size (∼100 bp) and a short ssDNA target (bowstring); during an experiment, a complementary ssDNA probe binds to and unbinds from this bow target. Combined with single-molecule FRET, DNA bows allow for high-throughput measurements of DNA hybridization and dehybridization kinetics in the low-force regime, using a conventional TIRF microscopy setup (Figure 2). Thus, this assay complements low-throughput, calibration-heavy tweezers (48,49). Using the DNA bow assay, we measured the hybridization and dehybridization rates of four DNA-DNA homoduplexes (lengths ranging from 8 to 9 bp) as well as their corresponding RNA–DNA heteroduplexes. Overall, the measured dehybridization (unbinding) rate monotonically increased with force with no clear sign of roll-over, and the measured hybridization (binding) rate also increased with force. In agreement with these experimental results, our simulations reveal that hybridization and dehybridization of short oligonucleotides transition through a maximally extended state, and as a result both processes are accelerated in the low force regime. We attribute the higher extension of the transition state to steric repulsion, which prevents the ssDNA overhangs of the nucleated duplex from coiling. Our simulations also reveal that the force-extension relations of bound, unbound, and transition states are linear, which enables derivation of simple equations for force-to-rate conversion. Our findings are consistent with those of a previous study (48) using optical tweezers and further reveal the extended nature of the transition state.

## MATERIALS AND METHODS

### Preparing DNA bows

**Figure 3.**
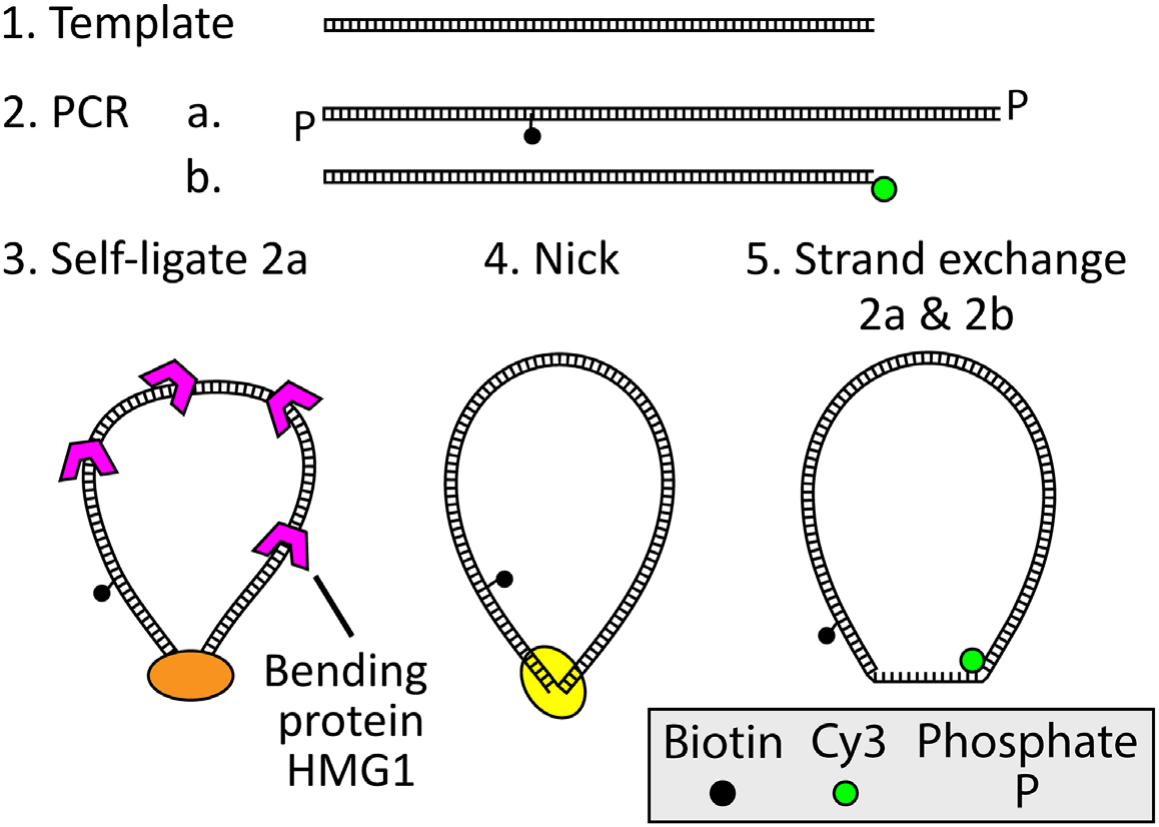
DNA bow construction. DNA bows were constructed in five steps. First, uniquely sized templates were generated with PCR from a common source. Using these templates, two sets of molecules (2a and 2b) were amplified with modified primers via PCR. DNA minicircles were then created from the phosphorylated 2a molecule set using protein-assisted DNA self-ligation. Afterward, DNA minicircles were purified and nicked on the unmodified strand. The final DNA bow constructs were finally constructed by exchanging the nicked strand of circularized 2a molecules with the Cy3-labeled 2b molecule.

DNA bow molecules were constructed and labeled with a FRET donor (Cy3) and a biotin linker in 5 steps (Figure 3): (1) template generation, (2) modifier incorporation, (3) circularization, (4) nick generation and (5) strand exchange. Most notably, DNA bending protein HMG1 was used to facilitate intramolecular ligation of short DNA molecules (50).

In Step 1, polymerase chain reaction (PCR) was used to create a set of seven different DNA templates with lengths ranging from 74 to 252 bp (Supplementary Figure S2, Supplementary Table S1), using yeast genomic DNA as the source. The PCR primers were designed such that all seven templates shared adaptor sequences at their ends. In Step 2, using these templates, two additional PCR reactions were performed to create two sets of molecules, with each reaction using modified primers that anneal to the adaptor regions of the template. The first reaction produced a set of molecules with phosphorylated 5′ ends and an internal biotin-dT label for surface immobilization, as well as a 15 bp extension, consisting of a 9 bp target segment flanked on both sides by (dT)_3_ spacers. The second reaction produced donor-labeled (Cy3) molecules with a sequence identical to the original templates, which is 15 bp shorter than the first PCR product. All oligonucleotides were purchased from Eurofins MWC Operon and Integrated DNA Technology. All PCR products in the first and second steps were inspected by gel electrophoresis and extracted using a PCR clean-up kit. In Step 3, we circularized the phosphorylated molecules. To increase circularization efficiency, molecules were briefly incubated at 15 nm with 0.75 μm DNA bending protein HMG1 (Sigma Aldrich) in T4 ligase buffer for 10 min. Afterward, T4 ligase was added and the reaction volume was incubated overnight at 15°C. The reaction was stopped via heat inactivation, after which T5 exonuclease was added to remove linear inter-molecular or nicked intra-molecular ligation products. Finally, Proteinase K was added to remove any protein leftovers. The remaining circular molecules were purified and concentrated using ethanol precipitation. In Step 4, the unmodified strand of our circular molecules was nicked using Nb.BbvCI in 1× CutSmart buffer (NEB). After circularization and nicking, the resulting product was visualized and purified on a native polyacrylamide gel (6%, 29:1 acrylamide to bis-acrylamide in 0.5× TBE buffer) , which appeared as a a single, isolated band as shown in Supplementary Figure S3. The bands were extracted using a simple ‘crush-and-soak’ method, and then concentrated using the same ethanol precipitation method as before. In Step 5, a strand-exchange reaction was performed, replacing the nicked strand on each circular molecule with the corresponding donor-labeled linear strand. Circular molecules were mixed with the donor-labeled linear molecules at a 4:1 ratio, briefly heated to 95°C, and gradually cooled down to 4°C.

### DNA bow assay

Microscope slides with pre-drilled holes and coverslips were cleaned by sonicating in deionized water, drying in a vaccuum chamber, and 5-minute etching in a plasma chamber. The cleaned slides and coverslips were then passivated with PEG (polyethylene glycol) to minimize nonspecific binding. After PEGylation, the flow cell was assembled by joining the slide and the coverslip with double-sided tape and epoxy glue. The flow cell interior was incubated with NeutrAvidin followed by 50 μL of 40 pm DNA bow solution. Each measurement began after perfusing 20 nm of ssDNA probe solution into the flow chamber. The temperature of the flow chamber was maintained at 22°C using an objective lens temperature controller. For each molecule, a high Cy3 signal (low FRET) indicates a DNA bow in the unbound state, while a high Cy5 signal (high FRET) indicates a DNA bow bound with the probe (Figure 2). Bound and unbound lifetimes of approximately ∼100 immobilized molecules were collected in each trial; 2-4 trials were performed for each bow size. All data was collected on an objective-based TIR microscope with an EMCCD camera (DU-897ECS0-BV, Andor). Frame times varied from 50 to 1000 ms, depending on the duplex sequence. The imaging buffer contained 100 mm NaCl, 100 mm Tris (8 pH), a triplet state quencher (1 mM Trolox), and the protocatechuic acid (PCA)/protocatechuate-3,4-dioxygenase (PCD) system (51). Using this system, photobleaching was negligible at all donor excitation power settings and camera acquisition times used in our experiments (Supplementary Figures S4 and S5).

### Data analysis

For each trial, time trajectories of FRET values were extracted from surface-immobilized molecules with in-house Matlab codes. Briefly, we calculated the FRET signal for each molecule from the background-subtracted intensities of the donor signal (*I*_*D*_) and the acceptor signal (*I*_*A*_) with *I*_*A*_/(*I*_*A*_ + *I*_*D*_). Next, we filtered FRET trajectories with a moving average, and used FRET signal thresholding to mark discrete transitions between the two FRET states. The dwell times in the bound (‘on’) state (high-FRET state) and the unbound (‘off’) state (low-FRET state) were collected from each FRET trajectory. The binding rate (*k*_on_) and the unbinding rate (*k*_off_) were calculated from the mean dwell times (τ) using *k*_on_ = ([*c*]τ_off_)^−1^ and $k_\mathrm{off}=\tau _\mathrm{on}^{-1}$, where [*c*] is the concentration of Cy5 labeled probes. Typically, ∼150 trajectories were used for each rate measurement.

### Estimating the tensile force exerted by a DNA bow

To estimate the tension exerted on the ssDNA bowstring, we treated the dsDNA arc as a worm-like chain. The force *f* exerted by a worm-like chain along its end-to-end direction at distance *x*_0_ can be calculated from the end-to-end distance (*x*) distribution *p*(*x*) of the chain according to


(2) $$\begin{equation*} f(x_0) = -k_BT\frac{\partial \log p(x)}{\partial x}\Big |_{x_0}. \end{equation*}$$

For *p*(*x*), we used an interpolated formula (Supplementary Equation S3), which is accurate for a wide range of bending stiffness values (52).

With our bow design, *x*_0_ also corresponds to the equilibrium extension of the ssDNA bowstring, and therefore its value will depend on both the bow size as well as whether the probe is bound to the complementary target segment. To find a realistic value of *x*_0_, we performed oxDNA2 simulations (53–55) for all possible combinations of bow size, target sequence, and probe state (bound or unbound). DNA bows bound to an RNA probe were not simulated; while oligomeric RNA-DNA duplexes have a slightly smaller helical rise (56), the overall effect that this difference would have on the force is negligible. Each MD simulation was run for *t* = 1.52 μs, using a time step of 15.2 fs. For each trajectory *n* = 1 × 10^5^ configurations were saved in 15.2 ps evenly spaced intervals. Using these saved configurations, we calculated the extension *x*, defined as the distance between the bases located at the terminal ends of the dsDNA bow and linked to the ssDNA target strand. The exact location of each terminal base was specified by its center of mass. Afterward, we calculated the mean extension ($\overline{x}$) and standard deviation σ(*x*) for each molecule’s *x* distribution, to estimate *x*_0_ and its associated uncertainty respectively (Supplementary Table S2, Supplementary Figure S6). The tensile force $f(\overline{x})$ was then calculated using Equation (2), and the uncertainty in the tensile force was estimated with σ(*x*) by propagation of error, using $\partial f(x)/\partial x\Big |_{\overline{x}}\cdot \sigma (x)$. Additional details regarding WLC parameters and oxDNA2 simulations are provided in Supplementary Materials (84).

We also considered how sequence dependent intrinsic curvature would affect the force values. For this purpose, we used the rigid base pair model with nonzero intrinsic values for roll and tilt (57). The ground-state conformations of all bow sizes predicted by this model are shown in Supplementary Figure S7A. To calculate the elastic forces exerted by these intrinsically curved DNA bows, we minimized the following multi-variable energy function using the gradient descent method:


(3) $$\begin{equation*} E\lbrace x_i\rbrace =\sum _{i=1}^{3N-3}\beta _i\left(x_i-x_{i,0}\right)^2+\kappa \left(r\lbrace x_i\rbrace -r_0\right)^2. \end{equation*}$$

This energy function represents the total energy of the DNA segment in the presence of a spring with stiffness κ that restrains the distance *r* between the two sticky ends to a small value *r*_0_. *x*_*i*_ is the roll, tilt, or twist rotation angles of each base pair step while β_*i*_ and *x*_*i*, 0_ are the corresponding stiffness parameter and ground-state angle, respectively. The force exerted on the target strand by the DNA arc can be estimated from the force exerted by the restraining spring evaluated with the set of values $\lbrace \tilde{x}_i\rbrace$ that minimizes *E*


(4) $$\begin{equation*} f\approx 2\kappa \left(r\lbrace \tilde{x}_i\rbrace -r_0\right). \end{equation*}$$

The minimization was initiated from a DNA conformation that is uniformly curved toward an arbitrary rotational register angle (58). We randomly varied this angle to sample different initial conformations. The mean and standard deviation of these forces are presented in Supplementary Figure S7B. Although the force values calculated this way do not account for the entropic fluctuations of the DNA arc, they are still similar to the values calculated from the full partition function in the short length regime.

### Observing the force-extension behavior of near-transition oligoduplexes

To measure the force-extension behavior of a nucleated oligoduplex near its binding or unbinding transition, we performed a series of MD simulations using the ‘mutual trap’ external force tool provided with oxDNA2. We simulated the target strand in four states: the ‘probe-bound’ state (9 bp), the ssDNA ‘probe-unbound’ state (0 bp), a transition state with 1 bp remaining at the 3′ end of the duplex, and a transition state with 1 bp remaining at the center. In the transition state simulations, the remaining terminal or middle base pair interaction was strengthened 10-fold, while all other base pairing interactions were set to zero. For all simulations, the ends of the target strand were connected by a harmonic spring with stiffness *k* = 57.1 pN nm^−1^ (1 simulation unit) and relaxed extension *x*_0_, such that the the tension *f* and extension *x* of the strand could easily be related using $f=-k\cdot (\overline{x}-x_0)$. Similar to our DNA bow simulations, the extension *x* was defined as the distance between the center of mass of each terminal base on the target strand. This definition includes 6 nucleotides flanking the probe-binding region to match the target strand in our DNA bow. For each state, we performed MD simulations for a small range of *x*_0_ values, such that the corresponding forces approximately spanned the force range of our DNA bows. For comparison, we plot the force-extension behavior of the target strand extended by a harmonic spring or a DNA bow in Supplementary Figure S8. Each simulation was performed for *t* = 1.52 μs using a time step of 15.2 fs. *n* = 10^5^ pairs of force and extension values were then calculated from configurations collected in 15.2 ps intervals evenly spaced across the MD trajectory.

### Sampling the free energy landscape of a melting duplex subject to weak tension

The free energy of a 9 bp oligoduplex during its binding or unbinding transition was sampled by performing virtual-move Monte Carlo simulations (VMMC) with oxDNA2. To capture how short duplex dynamics are coupled with force, we sampled the landscape using two distinct reaction coordinates: the extension *x* of the target strand, and the number of remaining base pairs *n*_bp_ in the probe-target duplex. The two-dimensional free energy landscape was then calculated using:


(5) $$\begin{equation*} G(x,n_\mathrm{bp}; f) = -k_\mathrm{B}T\log p_\mathrm{eq}(x,n_\mathrm{bp};f)+C. \end{equation*}$$

Here, *p*_eq_(*x*, *n*_bp_; *f*) is the probability of observing the given parameter values in equilibrium, and *C* is a constant independent of *x* and *n*_bp_ (59). Molecular configurations were binned along the *x* direction in 0.1 simulation unit (0.085 nm) intervals; *n*_bp_ values were defined as the number of remaining nucleotide pairs with hydrogen bonding energy less than –0.1 simulation units (1 *k*_B_T at 22°C). To ensure that all of the intermediate *n*_bp_ states were well-sampled and thereby accelerate transition reactions, we implemented umbrella sampling; for each base pair value *n*_bp_, a unique bias value *W*(*n*_bp_) was applied to the system’s Boltzmann distribution. *W*(*n*_bp_) was set to increase exponentially with *n*_bp_ (specific values are tabulated in Supplementary Table S3). After running a simulation, we calculated the equilibrium probability of each *n*_bp_ state using:


(6) $$\begin{equation*} p_\mathrm{eq}(x,n_\mathrm{bp};f) = \frac{p_{\mathrm{biased}}(x,n_\mathrm{bp};f)}{W(n_\mathrm{bp})}. \end{equation*}$$

For each simulation, we applied a unique constant tension value to the target strand. Force values ranged from 1 to 7 pN in 1 pN increments, fully spanning the range of our experimental assay. To more closely resemble our experiments, tensile forces were oriented along the instantaneous end-to-end vector of the target strand, rather than along an arbitrary spatial coordinate. Each simulation was performed for 2 × 10^9^ steps; additional simulation details and parameters are provided in the Supplementary Materials.

## RESULTS AND DISCUSSION

Using the DNA bow assay, we measured the binding and unbinding rates of a short DNA or RNA (8- or 9-nt) oligonucleotide to a weakly pulled complementary target strand (15 nt). The measured binding (*k*_on_) and unbinding (*k*_off_) rate constants thus reflect hybridization and dehybridization transitions of a short DNA homoduplex or DNA/RNA heteroduplex. Our DNA bow assay exploits the bending rigidity of dsDNA to generate small forces and is conceptually similar to the force clamp implemented with DNA origami (60) and a loop-based force transducer (61). An identical DNA construct has also been used in other studies (62,63). Our DNA bow assay offers unique advantages over other single-molecule force assays such as optical and magnetic tweezers in that (1) force measurements can be performed on many molecules in parallel, and (2) all molecules experience the same force, free of bead-dependent heterogeneity. We created 21 DNA bows in total (Supplementary Figure S2), including seven different dsDNA lengths (74, 84, 105, 126, 158, 210 and 252 bp) for the elastic arc segment and 3 unique sequences for the complementary segment of the ssDNA target. The DNA bow was further designed such that only the desired gapped DNA circle can generate the FRET signal from the surface upon probe binding (Supplementary Figure S9). While sharp bending is known to disrupt the helical structure of circular DNA by generating ‘kinks’, these deformations do not appear in circles larger than 84 bp (64). Therefore, kinking is expected to be negligible even for our smallest DNA bow size, which includes a flexible 15 bp ssDNA segment in addition to its 74 bp dsDNA arc. We thus treat the DNA arc as a simple worm-like chain to calculate the force exerted on the target strand (see Methods), which is in the range of 1.70–6.34 pN in the unbound state and 1.6–6.25 pN in the bound state. Although direct force calibration of our DNA bows is experimentally infeasible, a few observations validate the accuracy of our force calculation: (i) the zero-force extrapolation based on the calculated forces matches the experimental data obtained in the absence of force in our previous study (65) and our current study (Supplementary Figure S10) and (ii) the uncertainty in our force calculation due to sequence-dependent intrinsic curvature of DNA is insignificant (Supplementary Figure S7).

### Binding and unbinding rates versus force

**Figure 4.**
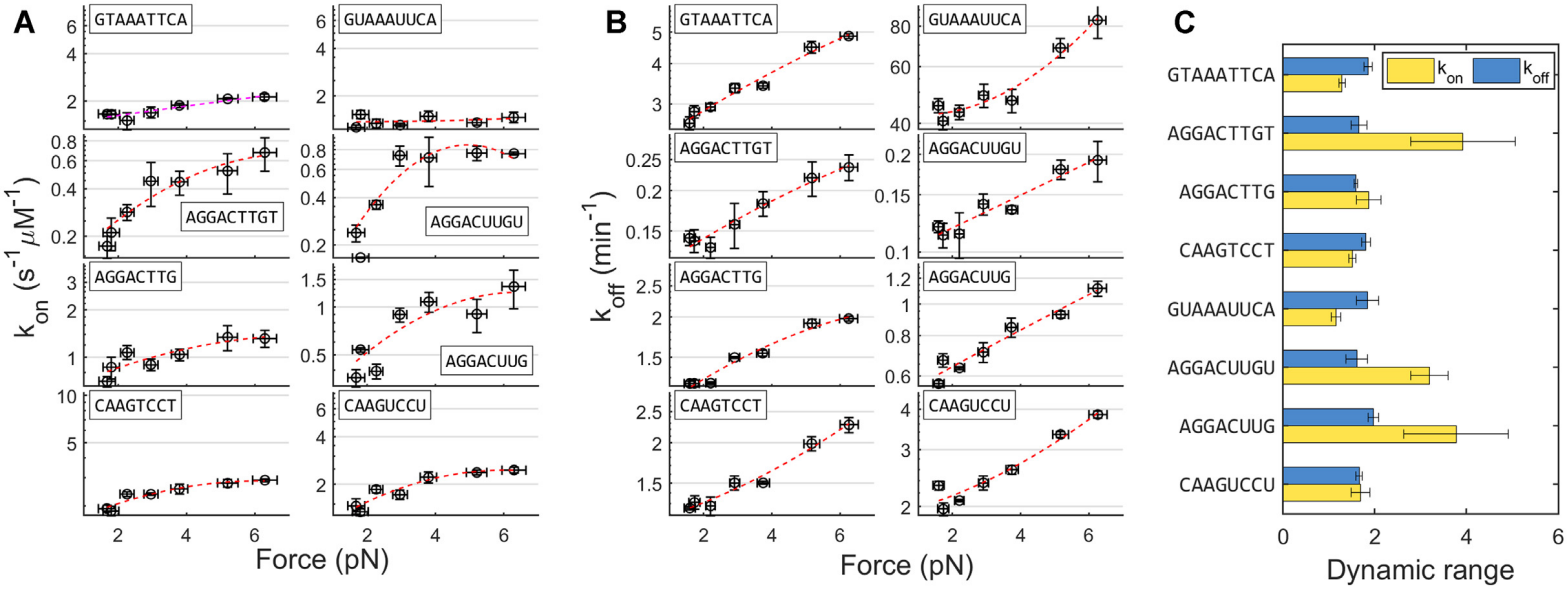
Experimental results. (**A**) Binding rate vs. force. The plots on the left (right) column are for DNA (RNA) probes. The y-axis is on a logarithmic scale over the same 5.8-fold change for all probe sequences. (**B**) Unbinding rate vs. force. The plots on the left (right) column are for DNA (RNA) probes. The y-axis is on a logarithmic scale over the same 2.4-fold change for all probe sequences. Vertical error bars for binding and unbinding rates represent the standard error of the mean. Going from top to bottom, the average number of molecules observed in each DNA probe trial was 178, 81, 189 and 145; the average number of molecules observed in each RNA probe trial was 159, 63, 207 and 107, respectively. Horizontal error bars were calculated using $\frac{\partial f(x)}{\partial x}\Big |_{\overline{x}} \cdot \sigma (x)$, where $\overline{x}$ and σ(*x*) are the mean and standard deviation of the bow’s end-to-end distance distribution. The fits based on our phenomenological model are shown in red. (**C**) The dynamic range of all measured rates. The dynamic range was obtained by dividing the rate at the highest force by that at the lowest force; the associated error was calculated by propagating the uncertainty in the underlying rates.

In Figure 4, we present the measured force dependence of *k*_on_ and *k*_off_ for four DNA-DNA duplexes (left column) and four RNA–DNA duplexes (right column). The scale of *y*-axis is set as logarithmic to aid comparison to Equation 1. Each RNA sequence is identical to a corresponding DNA sequence, except for T to U substitution. As shown in Figure 4A, *k*_on_ tends to increase with force over the measured force range. The relative increase in *k*_on_ is sequence-dependent: the increase is relatively large for AGGACTTGT but small for GTAAATTCA. The relative increase or dynamic range is quantified by taking the ratio of the rate at the highest force to that at the lowest force (Figure 4C). This sequence-dependence was also observed in RNA-DNA duplexes, with each heteroduplex approximately matching the behavior of its corresponding homoduplex. However, the sequence-dependence of the binding dynamic range is largely determined by the relative increase seen in *k*_on_ from 0 to 3 pN. Beyond this point, the slope of *k*_on_ decreases significantly, and appears to reach a plateau above 6 pN.

The most significant result from Figure 4A is that *k*_on_, the binding rate of the probe to its complementary target, becomes faster, not slower, as the tension in the target strand increases. This result stands in contrast to previous rates observed at higher forces, such as those observed for DNA hairpin folding (66–68), but is consistent with the result of a previous study (48) using a similar pulling geometry at low forces. By differentiating the logarithm of Equation 1 with respect to force, we can relate the slope of curves in Figure 4A to Δ*x*^‡^, which is the extension of the transition state (*x*^‡^) relative to the unbound state (*x*_u_):


(7) $$\begin{equation*} \frac{d\log {k_{\mathrm{\alpha }}(f)}}{df}=\frac{\Delta x^\ddagger (f)}{k_B T}. \end{equation*}$$

The overall non-negative slope in Figure 4A indicates that the transition state for hybridization is more extended than the unbound state (*x*^‡^ > *x*_u_) in the range of   2-6 pN.

The force dependence of *k*_off_ is shown in Figure 4B. Compared to *k*_on_, the dynamic range for *k*_off_ is somewhat uniform at 2-fold across all DNA-DNA and DNA-RNA duplexes (Figure 4C). The apparent slope is mostly positive except between a few points below 2 pN, which implies that the roll-over effect or catch-to-slip transition is negligible. According to Equation (7), the slope of curves in Figure 4B is proportional to Δ*x*^‡^ for dehybridization, which is the extension of the transition state (*x*^‡^) relative to the bound state (*x*_b_). From this, we conclude that the transition state for dehybridization is more extended than the bound state (*x*^‡^ > *x*_b_) in the range of 2–6 pN.

Since the first-order rate constant *k*_off_ is concentration-independent, it can be compared across different sequences. When compared at the same force, *k*_off_ was in the order of GTAAATTCA > AGGACTTG = CAAGTCCT > AGGACTTGT from fastest to slowest. When a single nucleotide was removed from the 3′ end of AGGACTTGT, *k*_off_ increased as expected from the weaker base pairing interaction. Between AGGACTTG and its reverse complement CAAGTCCT, *k*_off_ remains the same, which implies that for a DNA–DNA homoduplex, *k*_off_ is similar regardless of which strand is subject to tension. *k*_off_ for RNA-DNA duplexes (Figure 4A, right) similarly showed a strong sequence-dependence, in the order of GUAAAUUCA>CAAGUCCU>AGGACUUG>AGGACUUGU. In two cases (AGGACUUGU and AGGACUUG), RNA-DNA heteroduplex was longer-lived than its homoduplex counterpart, but in the other two (GUAAAUUCA and CAAGUCCU), DNA-DNA homoduplex was longer-lived.

### Thermodynamic stability

From the individual rate constants, we can calculate the standard free energy difference (Δ*G°*) between the bound and unbound states according to


(8) $$\begin{equation*} \Delta G^\circ = k_\mathrm{B}T \log \frac{k_\mathrm{on}[c_0]}{k_\mathrm{off}} \end{equation*}$$

where [*c*_0_] is 1 m. In this definition, Δ*G*° is more positive for a more stable duplex. In Supplementary Figure S11, we compare Δ*G°* calculated using *k*_on_ and *k*_off_ measured at the lowest force with $\Delta G_\mathrm{NN}^\circ$ estimated using a nearest-neighbor (NN) thermodynamic model (69,85,86). Most sequences are significantly more stable than the model predicts, showing at least a 2 *k*_B_*T* difference. This increased stability can be attributed to two major factors. First, the terminal bases of the duplex will stack with the adjacent unpaired bases in the gaps, which has been shown to provide ∼1 *k*_B_*T* per end interaction in 8 bp DNA duplexes (70). Dangling nucleotides beyond these adjacent bases have also been shown to stabilize the duplex (71,72), albeit to a lesser degree (73). Second, the DNA and RNA probes used in this experiment were labeled with a Cy5 dye on the 5′ end, which will also stabilize short DNA duplexes by 2 *k*_B_*T* (74). The stabilizing effects of dangling-base interactions and 5′ dye labeling are additive (74). When accounting for these two factors, we find that the nearest-neighbor model prediction matches Δ*G°* more closely.

In Supplementary Figure S12, the free energy difference Δ*G°* is plotted against force. Because both rates change in the same direction in response to force, the force dependence of Δ*G°* is somewhat dampened. Except for AGGACTTGT and its RNA counterpart, Δ*G°* changes little, albeit with some scatter. In comparison, the force-dependence of Δ*G°* of AGGACTTGT and AGGACUUGU shows a monotonic increase up to 3 pN and afterward plateaus, varying by less than 0.5 *k*_B_*T*. Substituting Equation (1) for *k*_on_ and *k*_off_ into Equation (8), we obtain


(9) $$\begin{equation*} \Delta G^\circ \left(f\right) = \Delta G^\circ \left(0\right)-\int _0^f\left(x_u(f^\prime )-x_b(f^\prime )\right)df^\prime , \end{equation*}$$

which can be used in conjunction with the force-extension relations of ssDNA and dsDNA to calculate Δ*G°*(*f*). Using the Marko-Siggia force-extension formula (Supplementary Equation S1) for ssDNA and dsDNA with respective parameters reported in Whitley et al. (48), we calculated Δ*G°*(*f*) and fitted this function to the measured Δ*G°*(*f*) for each sequence using Δ*G°*(0) as the only fitting parameter. As shown in Supplementary Figure S12A, the calculated Δ*G°*(*f*) is a concave function with a maximum near 4.25 pN, where the extensions of ssDNA and dsDNA become identical. The fits compare better for some sequences than others, which motivates us to include sequence-dependent parameters into our model, as will be discussed below.

### The physical nature of the transition state(s)

**Figure 5.**
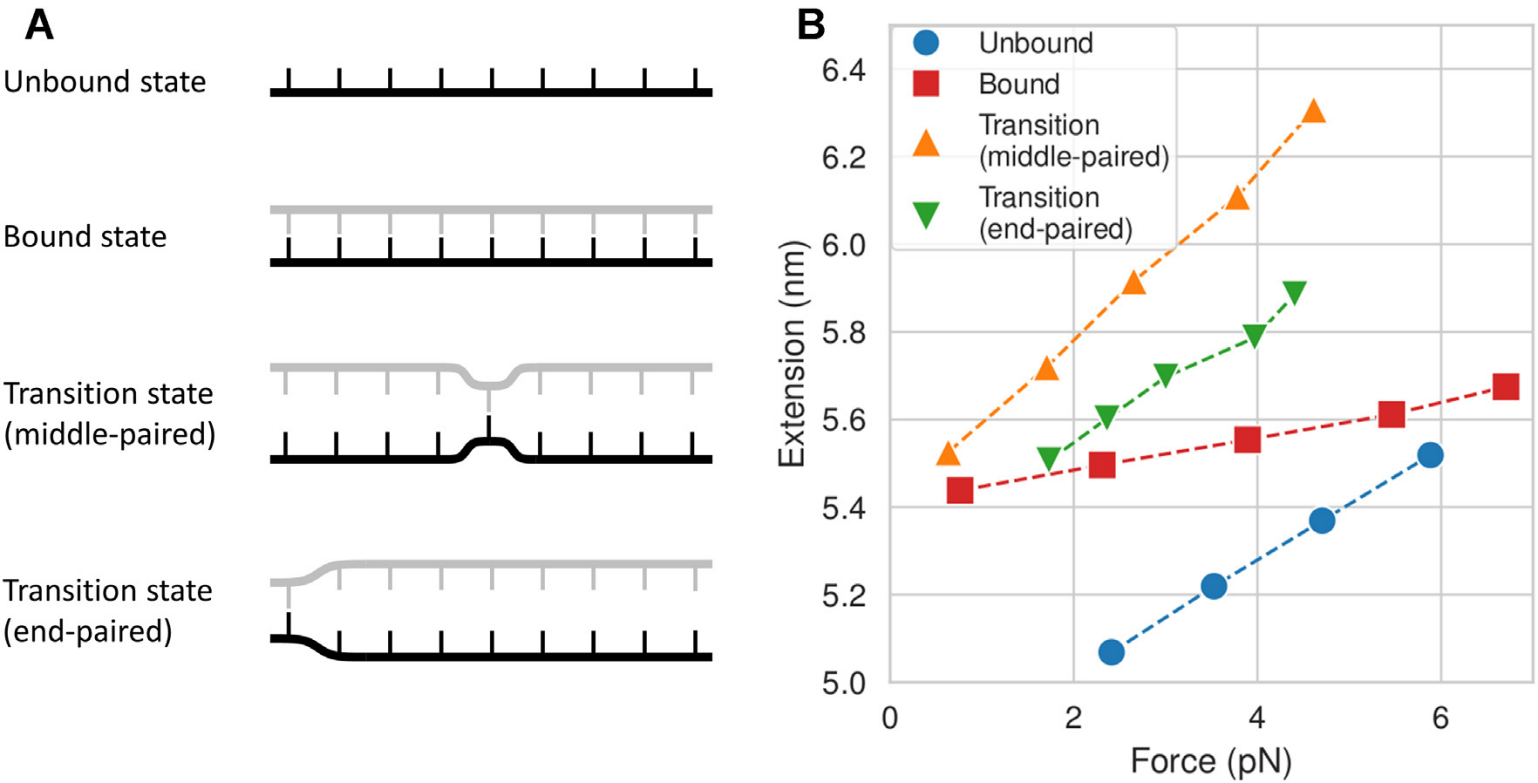
Simulations of force-extension relations. (**A**) Schematic of target strand (black) in four unique states: the unbound state, the bound state, the transition state with one base pair in the middle, and the transition state with one terminal base pair. (**B**) Force–extension curves of target strand in each state. We note that the bound state contains 3-nt ssDNA overhangs, and therefore appears more extensible than dsDNA.

Our DNA-bow experiments show that in the force range of 2–6 pN, both the binding and unbinding rates increase with force. The fact that weak force increases the accessibility of the transition state implies that the transition state is more extended than the two observable states, bound and unbound. For simplicity, we assume that the transition state is a state where a single base pair is formed or to be broken between the target strand and its complementary probe. To rationalize our experimental results, we obtained the force-extension curves of bound, unbound, and two representative transition states (Figure 5A) from oxDNA2 simulations. Since the transition state is too transient to be analyzed in a normal dynamics simulation, we stalled the system near this state by turning off all base pairing interaction except in one central or terminal base pair, whose pairing interaction was strengthened 10-fold. For all states, we observed spring-like force-extension behavior, as expected for short duplexes subject to small force. Moreover, as shown in Figure 5B, we find that both the end-paired and the middle-paired transition states are more extended than the unbound state (ssDNA) and the bound state (dsDNA) over the force range of 2–6 pN. Hence, our simulation results are consistent with the measured force-dependence of both *k*_on_ and *k*_off_.

At first thought, it is not obvious why the transition state, which is a mixed state of ssDNA and dsDNA (75), is more extended than the bound and unbound state, which are dsDNA and ssDNA, respectively. We reason that ssDNA segments in the transition state always exist as a pair of dangling strands from a dsDNA segment, and therefore behave differently from ssDNA in the unbound state. We propose that these dangling strands adopt a more extended state than isolated ssDNA strands because of inter-strand steric repulsion (or excluded volume interaction). This idea is essentially identical to the thermodynamic argument for explaining the height of a polymer brush increasing with grafting density (76). In this analogy, dangling strands correspond to a much higher grafting density than a lone strand; therefore, in our case, ssDNA regions in the transition state are more extended than in isolation. Consistent with this idea, we also find that the middle-paired transition state with two pairs of dangling strands is more extended than the end-paired transition state with one pair of dangling strands (Figure 5B).

### Comments on roll-over or catch-to-slip transition

The roll-over effect where *k*_off_ becomes a convex function of *f* was postulated based on the idea that the transition state is a hybrid of ssDNA and dsDNA and that each obeys the force-extension formula for an ideal WLC (40,47) or a unique WLC with its own characteristics (48). The interpolation formula used in these models (Supplementary Equation S1), however, is not accurate for short chains (77,78). An alternative formula derived for short chains (79,80) places the crossover force at ∼1.8 pN (Supplementary Figure S1), which borders the force limit of our DNA bow assay. But even this formula (Supplementary Equation S1) is only appropriate in cases where fluctuations orthogonal to the pulling axis are small, which may not be satisfied by ssDNA in the unbound state or the transition state at small forces. Hence, we used oxDNA2 simulations to directly obtain the force-extension curves of the bound, the unbound, and the transition state. As shown in Figure 5B, all states exhibit linear force-extension behavior with a constant slope and a nonzero vertical intercept, and the transition state is more extended than both the bound and the unbound states. A crossover in extension between the bound and the transition state, which is a requirement for the roll-over (Equation 7), is not apparent above 2 pN. However, our study does not completely eliminate the possibility of a roll-over. First, our DNA bow assay cannot probe forces lower than 1.5 pN. In this range, we find that the extension of the transition state can become shorter than that of the bound state (Figure 5). Second, the roll-over effect is predicted to be more pronounced for longer oligonucleotides (47). A more thorough test of this model thus requires measuring the dehybridization rate of oligonucleotides longer than 10 nt, which is extremely slow (∼h^−1^). Therefore, the roll-over effect, if any, would only exist on a time scale too slow to bear physiological or practical significance.

**Figure 6.**
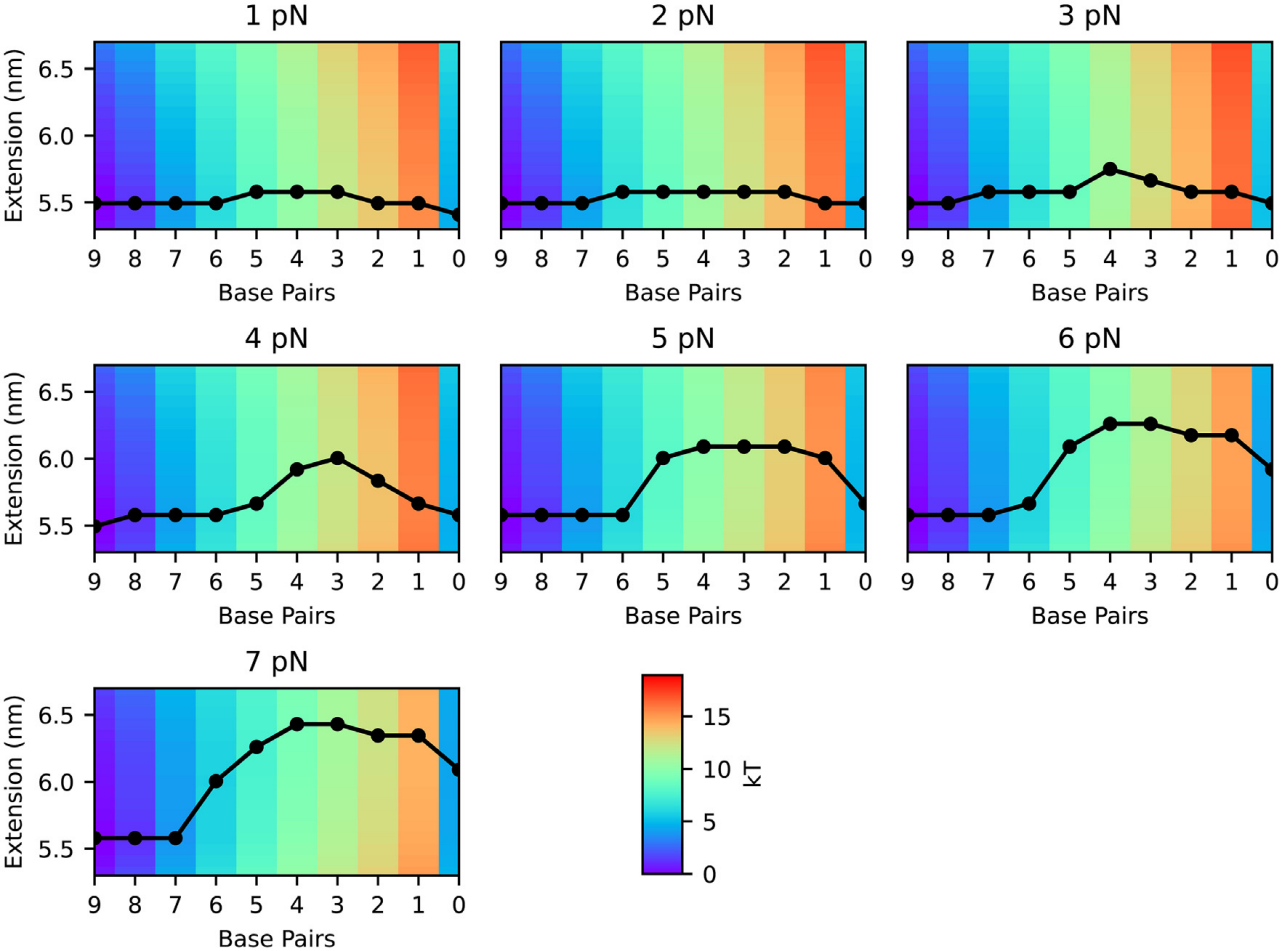
Heat maps of the free energy Δ*G*(*x*, *n*_bp_; *f*) surface as a function of molecular extension *x*, the number of base pairs between the target and probe strands *n*_bp_, and the tensile force *f*. Each black dot marks the extension value with the lowest energy for each base pair step. Probabilities for all combinations of *x*, *n*_bp_ and *f* were obtained by performing umbrella sampling simulations; the resulting free energy Δ*G* was then calculated using Equations (5) and (6).

To gain more insights into the force-extension relation during binding and unbinding, we sampled the free energy landscape of our probe-target duplex at several tension values using virtual-move Monte Carlo simulations. Free energy values *G*(*x*, *n*_bp_; *f*) were calculated using Equation (5) along two coordinates: the target-strand extension *x*, and the number of remaining base pairs *n*_bp_ in the duplex. To ensure that all intermediate states were well-sampled, we used umbrella sampling to bias the system toward high-energy melted states. The observed energy landscapes are represented as a series of heat maps in Figure (6). The heat maps show that *G*(*x*, *n*_bp_; *f*) varies markedly in the horizontal direction (*n*_bp_ than vertical (*x*), reaching maximum at *n*_bp_ = 1. To better visualize the landscape along *x*-direction, we also plot the most probable transition pathway for each landscape by connecting the extension values with the lowest energy for each base pair value ($\tilde{x}(n_\mathrm{bp})$) and present another series of heat maps by offsetting $G(\tilde{x}(n_\mathrm{bp}))$ to zero (Supplementary Figure S13). The most probable extension at *n*_bp_ = 1, which we assume to be the transition state, is always greater than or equal to that at *n*_bp_ = 9 or the bound state. Similarly, the extension at *n*_bp_ = 1 is greater than that at *n*_bp_ = 0 or the unbound state. These results are consistent with our result obtained from a different simulation in Figure 5.

### Quantitative models for force-dependence of rates

In our DNA bow assay, the tensile force is applied to the 3′- and 5′-ends of the same strand. In this case, base pairs experience the effect of force indirectly in the form of shear stress (81). This is in contrast to DNA unzipping experiments (82) where base pairs directly experience a rupture force in a sequential manner. As shown in Figure 6, the shear geometry imposes a weak coupling between *n*_bp_ and *x*: a decrease in *n*_bp_ does not always lead to a monotonic increase in *x*. Hence, different states along the reaction path cannot be fully specified with *x* only. Instead, states must be defined with respect to *n*_bp_.

The linear force–extension relations (FER) of states with different *n*_bp_ obtained in Figure 5 thus correctly represent the bound, unbound, and transition states. Based on these results, we assume that FERs of any sequence can be expressed as


(10) $$\begin{equation*} x_\alpha = f/a_\alpha +b_\alpha , \end{equation*}$$

where *a*_α_ and *b*_α_ are the stiffness constant and relaxed extension of the bound (α = *b*), unbound (α = *u*) or transition state (α = ‡). Deviation from this linear relationship is expected to be noticeable above 6 pN as observed in a previous study (48). Substituting these relations into Equation (1), we can obtain the analytical force dependence of *k*_on_ and *k*_off_. For each sequence, we simultaneously fitted two curves to the measured *k*_on_ and *k*_off_ using six parameters: *a*’s and *b*’s for unbound and transition states and *k*_*b*_(0) and *k*_*u*_(0), which are the zero-force rates. For simplicity, the extension of the bound state is fixed to 0.34 × *n*(nm), independent of force and sequence. The fitted curves are shown in Figure 4 in good agreement with the data, and the six parameters for each sequence are listed in Supplementary Table S4. The corresponding FERs of each sequence are shown in Supplementary Figure S14, where we find that: (i) FER of the unbound state (ssDNA) exhibits the strongest sequence dependence, and (ii) for most sequences, the extension of the transition state changes very little with force. Δ*G*°(*f*) calculated by taking the ratio of the two fitted curves also match the measured Δ*G*°(*f*) well (Supplementary Figure S12B). For comparison, we also tried fitting our data using the model by Whitley *et al.* (48). In this model, all three states are assumed to obey the Marko-Siggia FER (Supplementary Equation S1) with their own set of persistence length (*P*) and contour length (*L*). In this fitting, we fixed *P*_*u*, *b*_ and *L*_*u*, *b*_ to the values reported in Whitley et al.(48), but allowed *P*_‡_ and *L*_‡_ to vary for each sequence. The results from this fitting are presented in Supplementary Figure S15, in good agreement with the data. Moreover, *a*_‡_ (Supplementary Table S4) and *P*_‡_ (Supplementary Table S5) extracted from the two different models are well correlated, indicating their mutual consistency.

The extracted stiffness parameter *P*_‡_ of the transition state varies markedly from sequence to sequence. The sequence dependence of *P*_‡_ may reflect the variable location of the last remaining base pair in the transition state among different sequences. In support of this idea, the end-paired and the middle-paired transition states exhibit different slopes in the force-extension curve (Figure 5). The number of base pairs in the transition state may also differ among sequences, further contributing to variable *P*_‡_. Our assumption of sequence independent *P*_*u*_ in the models also likely yields an inflated sequence dependence of *P*_‡_. More extensive MD simulations with various sequences in both the unbound and the transition states are needed to test these possibilities.

Although both models discussed above explain our data well, our model based on the linear FER (Equation 10) allows us to derive a simple expression without numerical integration. If we assume that *x* scales linearly with the number of nucleotide units (*n*), FER can be expressed in terms of stiffness constant (κ) and relaxed extension (*x*_0_) of a single-nucleotide as


(11) $$\begin{equation*} x = n\left(1/\kappa \right)f+nx_0. \end{equation*}$$

By rescaling the FERs of eight different sequences by the corresponding *n* and averaging them, we can extract sequence-averaged κ and *x*_0_ for the transition state (‡) and the unbound state (*u*): κ_‡_ = 355.3 pN · nm^−1^, *x*_0, ‡_ = 0.39 nm, κ_*u*_ = 30.7 pN · nm^−1^, and *x*_0, *u*_ = 0.21 nm. Since we assume dsDNA to be almost inextensible, κ_*b*_ ≫ 1 and *x*_0, *b*_ = 0.34 nm. Integrating these universal FERs according to Equation (1), we obtain


(12) $$\begin{equation*} k_\mathrm{on}(f)\approx k_\mathrm{on}(0) \exp \left[\frac{n}{k_\mathrm{B}T}\left\lbrace \left(\frac{1}{\kappa _\ddagger }-\frac{1}{\kappa _u}\right)\frac{f^2}{2}+\left(x_{0,\ddagger }-x_{0,u}\right)f\right\rbrace \right], \end{equation*}$$

(13) $$\begin{equation*} k_\mathrm{off}(f)\approx k_\mathrm{off}(0) \exp \left[\frac{n}{k_\mathrm{B}T}\left\lbrace \left(\frac{1}{\kappa _\ddagger }\right)\frac{f^2}{2}+\left(x_{0,\ddagger }-x_{0,b}\right)f\right\rbrace \right]. \end{equation*}$$

Since the transition state is relatively stiff, *k*_off_(*f*) is well approximated by the Bell’s formula. It is noteworthy of mentioning that these equations are essentially identical to Equation (7) of Suzuki *et al.* (83) which was derived by averaging the two dimensional energy landscape *G*(*x*, *n*_bp_) over all *x*.

## CONCLUSION

DNA often experiences tension through passive or active mechanisms. In the presence of 5 pN of force, DNA polymer models predict that dsDNA and ssDNA have a similar extension, which can lead to a nontrivial force dependence of hybridization and dehybridization rates. Previous force spectroscopy techniques, however, are not optimal for investigating this force dependence due to limited throughput. In this study, we developed a DNA bow assay, which can exert 2–6 pN of tension on a ssDNA target and report on its hybridization and dehybridization via smFRET. In this force range, we found that both the hybridization and dehybridization rates increase with force, which indicates that the transition state has a longer extension than its ssDNA and dsDNA counterparts. Coarse-grained simulations reveal that hybridization and dehybridization proceed through a maximum free energy state where a single base pair is formed between the target and probe strands. Consistent with the experimental results, the simulations also show that this maximum free energy state, which we identify as the transition state, is more extended than the fully paired or unpaired state. We propose that the greater extension of the transition state is due to steric repulsion that precludes ssDNA overhangs from adopting random coil configurations. To conclude, we present a simple force-to-rate conversion formula based on linear force–extension relations.

## DATA AVAILABILITY

All data presented in this manuscript can be made available upon request from the corresponding author.

## Supplementary Material

gkad118_Supplemental_File
