# Supplementary material for: Weak tension accelerates hybridization and dehybridization of short oligonucleotides
Source: Nucleic Acids Res. 2023 Mar 3;51(7):3030–40. doi: 10.1093/nar/gkad118 (PMC10123125; doi:10.1093/nar/gkad118)
Supplement: gkad118_Supplemental_File [file gkad118_supplemental_file.pdf]

SUPPLEMENTARY MATERIAL

Force-extension curves of worm-like DNA

In the limit of $L \gg P$, Marko and Siggia derived an interpolation formula (MS formula) for the relationship between force (f) and extension (x) of a worm-like chain (WLC) [77]:

$$\frac{fP}{k_{\mathrm{B}}T} = \frac{x}{L} + \frac{1}{4\left(1 - x/L\right)^2} - \frac{1}{4} \tag{S1}$$

where P is the persistence length, and L is the contour length. It is convenient to define the contour length per nucleotide, $h = L/N$. The accuracy of this formula can be increased with additional terms [78]. Whitley et al. [48] and Guo et al. [46] modeled ssDNA and dsDNA as WLCs and also attempted modeling the transition state as a chimeric DNA of ssDNA and dsDNA or a WLC with its own unique P and L. Using 53 nm and 0.34 nm for P and b of dsDNA, and 1.32 nm and 0.6 nm for P and b of ssDNA in Equation S1 and inverting it, we can obtain x as a function of f (top, Supplementary Figure S1).

The extensions of ssDNA and dsDNA are predicted to cross over at $f \approx 4.3$. A different formula that is more correct for short WLC is derived by Keller et al. [79] and Hori et al. [80]. In this formula, x is expressed as a function of f:

$$x = L - \frac{k_{\mathrm{B}}T}{2f}\left(L\sqrt{\frac{f}{Pk_{\mathrm{B}}T}}\coth\left(L\sqrt{\frac{f}{Pk_{\mathrm{B}}T}}\right) - 1\right). \tag{S2}$$

Force-extension curves of ssDNA and dsDNA obtained from this formula are shown at the bottom of Supplementary Figure S1. The crossover force is ~ 1.8 pN, markedly lower than predicted by the MS formula.

oxDNA2 simulation parameters

For all oxDNA2 simulations, the buffer conditions were specified to be identical to our experiments (100 mM salt concentration and 22 °C). MD simulations were equilibrated for 50000 time steps before configurations were saved into output trajectories [59]. All MD simulations used an Andersen-like thermostat [84], where the system was propagated according to Newton's equations for N_{Newt} time steps using Verlet integration; afterward, the system was assigned new velocities from Maxwell-Boltzmann distribution such that the

resulting diffusion coefficient was equal to a specified value D. For all DNA bow simulations and force-extension simulations, $N_{\mathrm{Newt}} = 103$ and $D = 2.5$. For VMMC simulations, the maximum translational and rotational displacements were set to 0.1 and 0.2 respectively. All VMMC simulations were equilibriated for 10^5 steps before collecting statistics.

Estimating the end-to-end distance radial probability distribution of DNA bows

To estimate the tensile force f exerted by each DNA bow size (Equation 2), we used the following interpolation formula [52] to estimate the radial probability distribution $p(x)$ of a wormlike chain

$$p(x') = 4\pi x'^{\,2} \cdot J_{SYD} \cdot \left(\frac{1 - cx'^2}{1 - x'^2}\right)^{5/2} \exp\left(\frac{\sum_{i=-1}^{0}\sum_{j=1}^{3} c_{i,j}\kappa^i x'^{2j}}{1 - x'^2}\right) \times \exp\left(\frac{-d\kappa ab(1+b)x'^2}{1 - b^2x'^2}\right) I_0\left(-\frac{d\kappa ab(1+b)x'^2}{1 - b^2x'^2}\right), \tag{S3}$$

where

$$a = 14.054, \quad b = 0.473, \quad (c_{i,j})_{i,j} = \begin{pmatrix} -3/4 & 23/64 & -7/64 \\ -1/2 & 17/16 & -9/16 \end{pmatrix}.$$

This formula accurately models $p(x)$ for a large range of stiffness values ($\kappa = P/L$, where P and L are the persistence and contour lengths of the dsDNA elastic arc, respectively) as well as a wide range of normalized end-to-end distance values ($x' = x/L$). For this calculation, we assumed the values $P = 53\,\mathrm{nm}$ and $h = 0.34\,\mathrm{nm}$, where h is the contour length per nucleotide $b = L/N$ Therefore, Equation S3 can be used with Equation 2 to estimate the force exerted by all bow sizes, whose stiffnesses range from $\kappa = 0.6$ to κ=2.1, and whose end-to-end distance values range from $x = 0.06$ to $x = 0.21$.

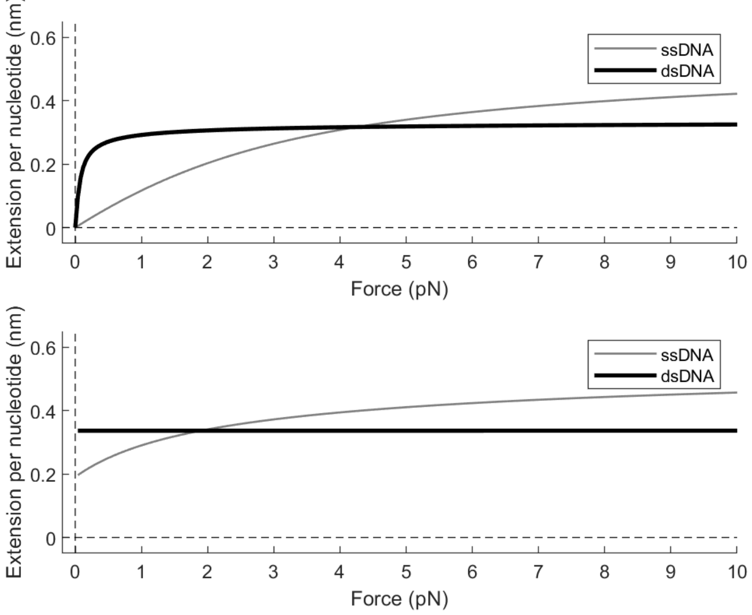

Supplementary Figure S1. Force-extension curves of ssDNA (light gray) and dsDNA (dark gray) based on Equation S1 (top) and Equation S2 (bottom). The location of the crossover point depends substantially on the approximation.

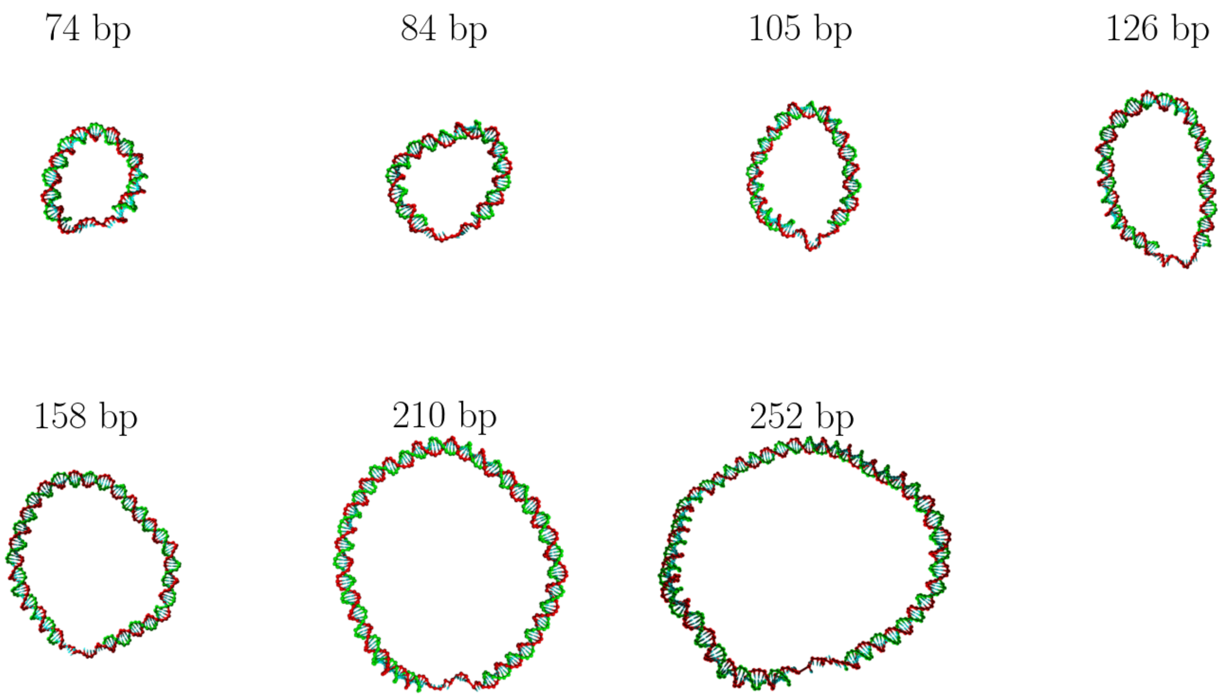

Supplementary Figure S2. Sample oxDNA2 configurations of all experimentally measured DNA bow sizes. Each configuration depicts the DNA bow in its unbound state. The label above each molecule specifies the length of the dsDNA bow arc (in base pairs). All constructs feature a 15 nt ssDNA strand containing a 9 nt region targeted by an 8-9 nt probe.

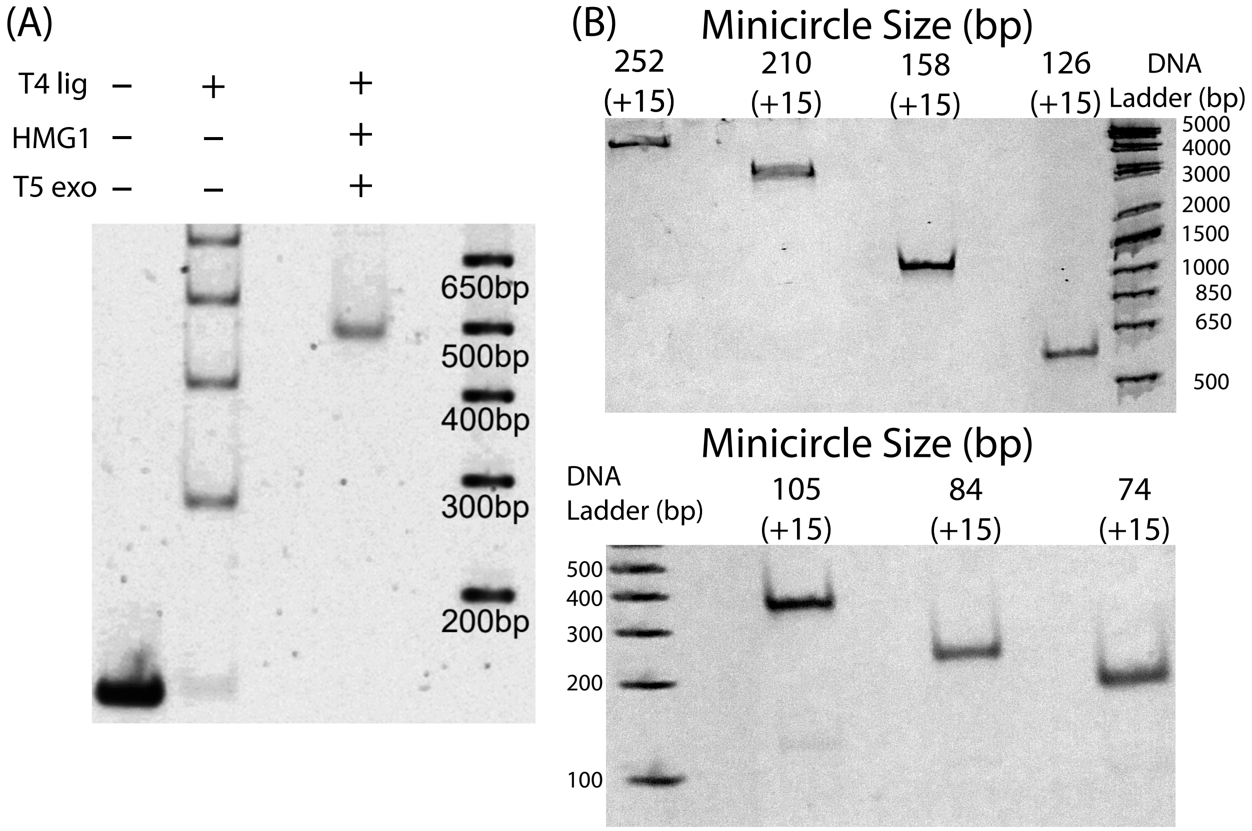

Supplementary Figure S3. **(A)** Linear DNA molecules with phosphorylated 5' ends were bent with bending protein HMG1 and self-ligated. T5 exonuclease was then added to digest unwanted polymer fragments. **(B)** The remaining circular DNA was then purified with ethanol precipitation and nicked on the unmodified strand with Nb.BbvCI. Nicked minicircle bands were analyzed and extracted using polyacrylamide gel electrophoresis (6%, 29:1 acrylamide to bis-acrylamide in 0.5x TBE buffer). Note that the total minicircle size includes the 15 bp target strand segment, which is not included in the bow arc length (74 bp to 252 bp. After each circle size was inspected via PAGE, DNA minicircles were extracted overnight via "crush-and-soak" and concentrated with ethanol precipitation.

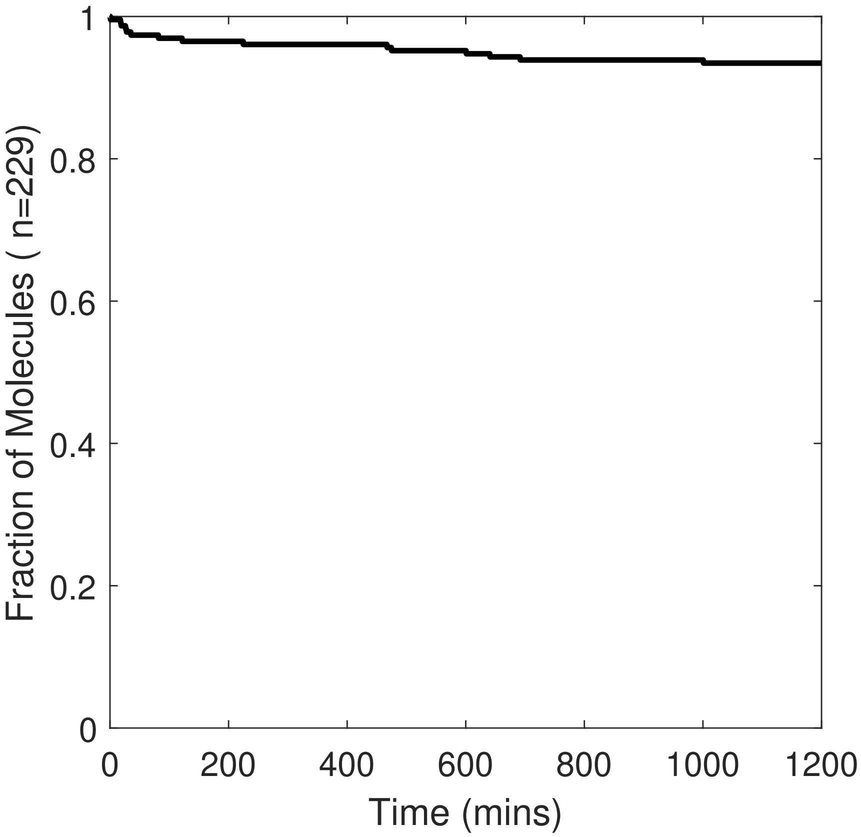

Supplementary Figure S4. Surviving fraction of fluorescent Cy3 molecules over time. Photobleaching time was defined as the first instant at which the Cy3 intensity trace fell below a threshold cutoff value. The threshold value was selected by analyzing the intensity histogram of all observed traces and choosing the minimum probability intensity value separating the bleached and unbleached states. Control molecules were assembled by annealing Hairpin 1 (see Table S1) to the Reverse Cy3 primer, using the same "heat and cool" annealing protocol as our original experiments. Control trials were performed using the same imaging buffer and maximum laser power (3 mW) of our original experiments.

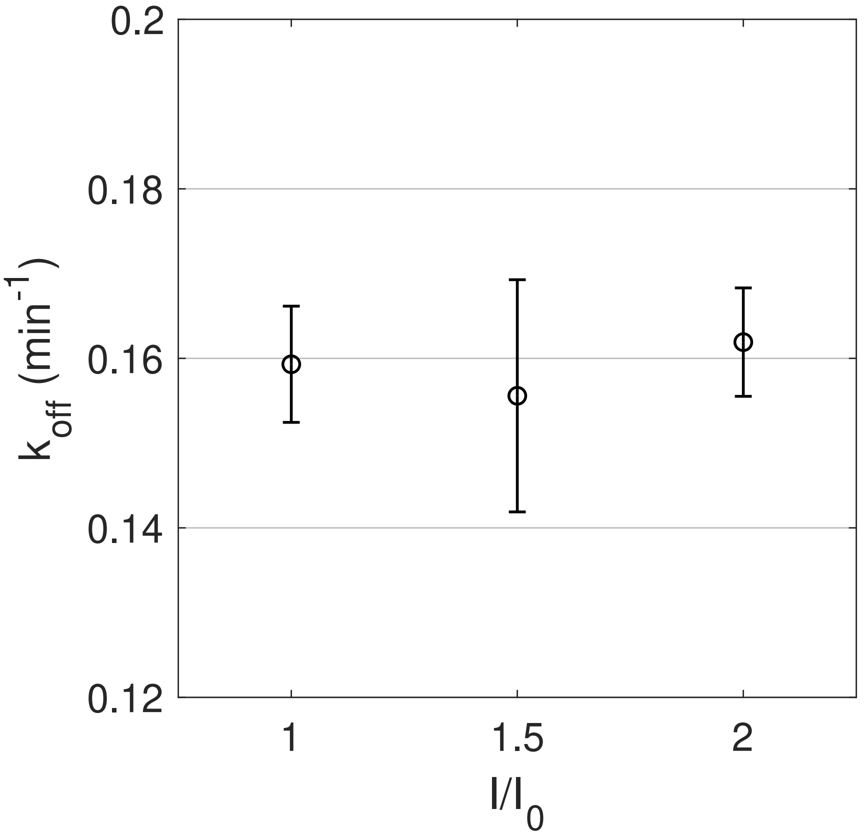

Supplementary Figure S5. Unbinding rate vs Cy3 laser power intensity. The effects of Cy5 photobleaching were tested using the same linear construct as in Supplementary Figure S4. Additionally, we added P2-RNA to the imaging buffer (the probe with the longest observed bound dwell time), to test whether laser intensity affects the unbinding rate. Three power settings were tested, including the original maximum value (3 mW) of our original measurements, as well as 2 larger settings: 4.5 mW and 6 mW. Two trials were performed for each power setting.

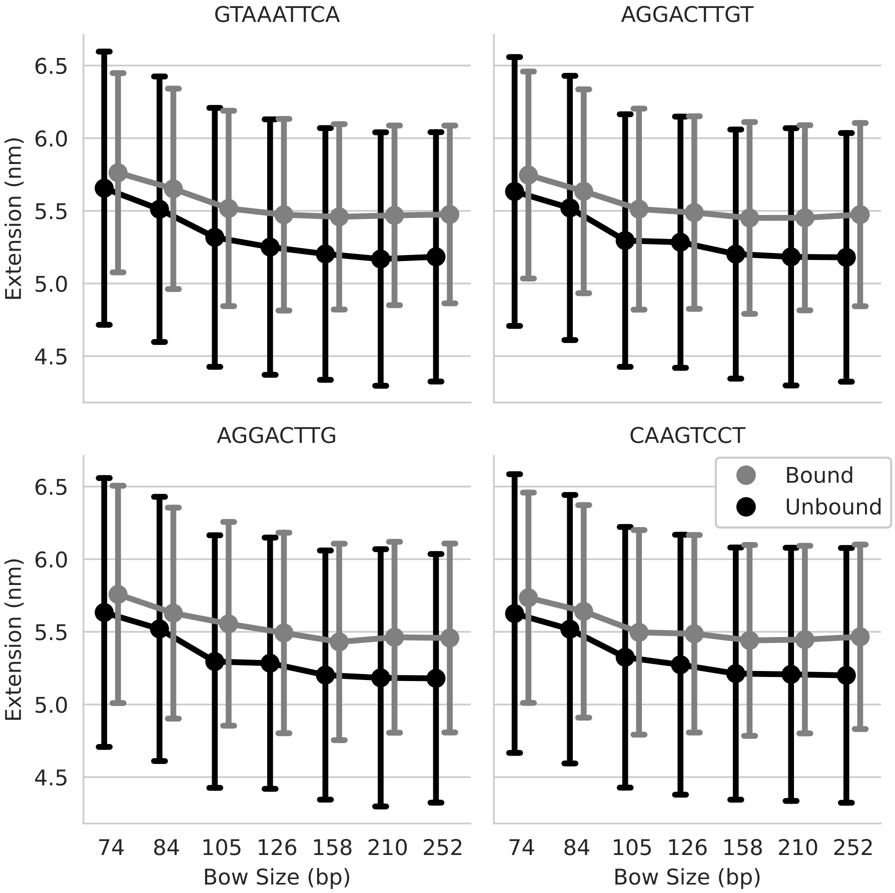

Supplementary Figure S6. Mean ($\overline{x}$) and standard deviation values ($\sigma(x)$) of the end-to-end distance x for each DNA bow size, in both the probe-bound and probe-unbound states. Each MD simulation was performed for 1×10^8 steps with $dt = 15.2\,\mathrm{fs}$, totaling $t = 1.52\,\mu\mathrm{s}$. Extension values were measured every 1000 steps, collecting $n = 1 \times 10^5$ values in total.

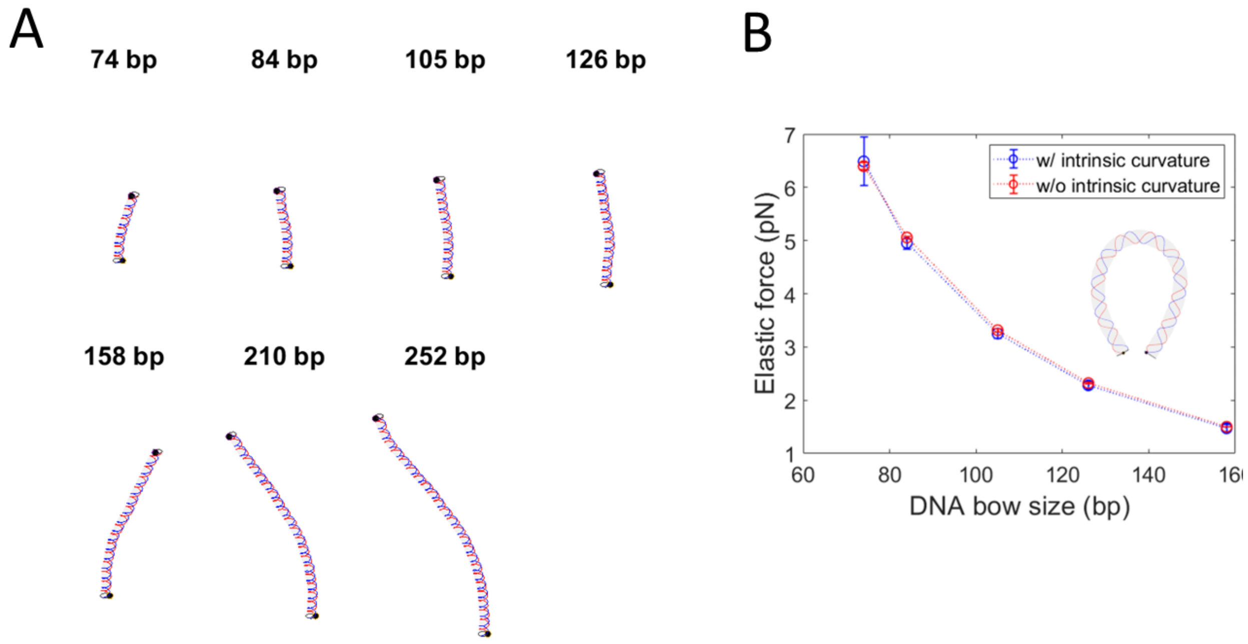

Supplementary Figure S7. The effect of intrinsic curvature on force calculation. **(A)** Ground state conformations of all DNA bow duplex sequences. Conformations were found using the intrinsic values and stiffnesses of dinucleotide roll and tilt tabulated by Zuiddam et al. [57]. **(B)** Elastic force calculations of all DNA bow sequences with and without intrinsic curvature. The blue curve uses intrinsic roll and tilt parameters of base pair steps that change with sequence. For the red curve, these intrinsic values are set to zero. The inset shows a typical minimum energy conformation.

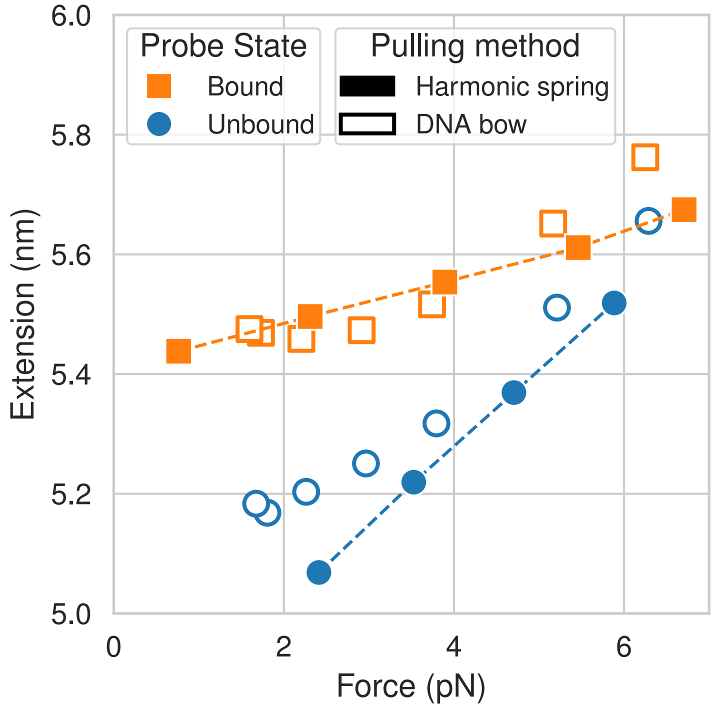

Supplementary Figure S8. Force-extension behavior of the 17 nt target sequence T1 in both its probe-bound and probe-unbound states, extended by either a harmonic spring (filled markers) or a DNA bow (open markers). For the harmonic spring pulling method, we used the "mutual trap" external force tool provided with oxDNA to connect the terminal bases of the target with a weak spring.

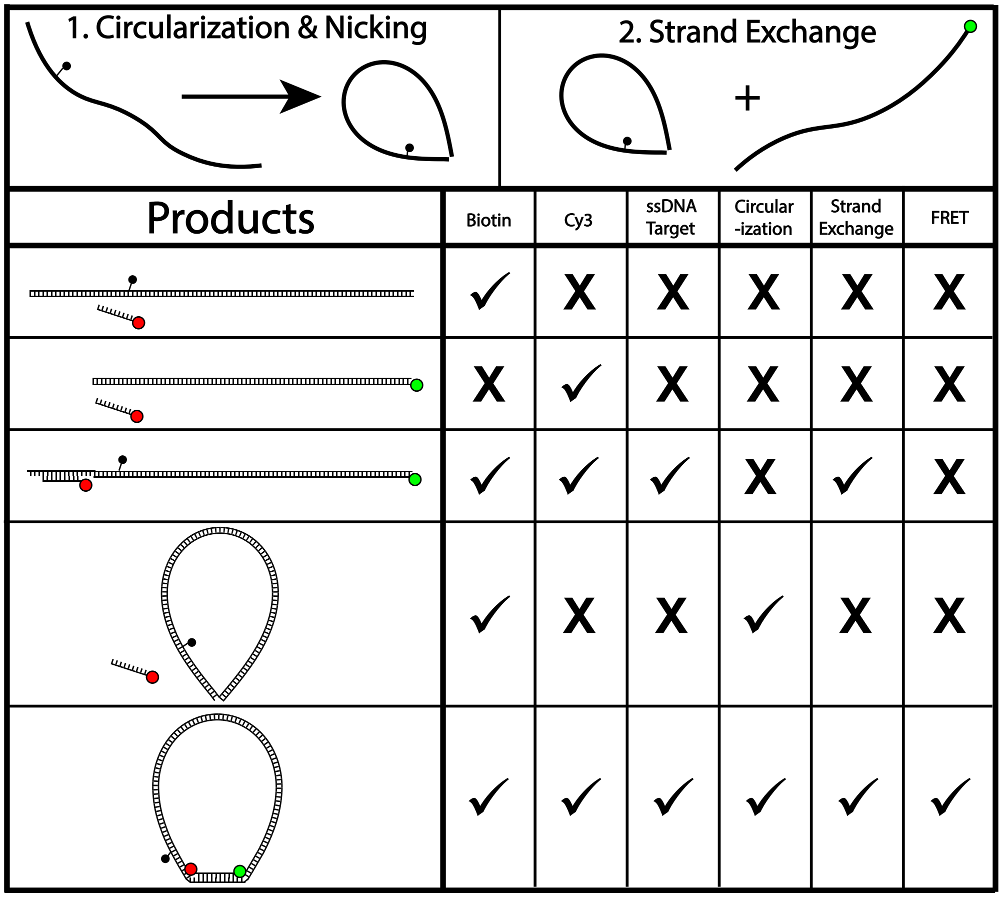

Supplementary Figure S9. Possible products created during bow construction. Nicked circular products were purified and mixed with Cy3-labeled linear molecules at a 1:4 ratio. The unmodified nicked strand is replaced via a strand exchange reaction which consists of heating the mixture to 95 °C and cooling to 4 °C gradually. By design, only the desired product (bottom row) is capable of generating a FRET signal. Incorrect purification of nicked circular strands (step one) would yield linear products during strand exchange; among these products, the target is either too far from the Cy3 dye to generate a FRET signal upon probe binding, or the target is absent entirely. Circular molecules that do not replace the unmodified strand during the strand exchange reaction (step two) are not donor-labeled, nor do they have an exposed acceptor-probe target, and therefore also cannot generate a FRET signal.

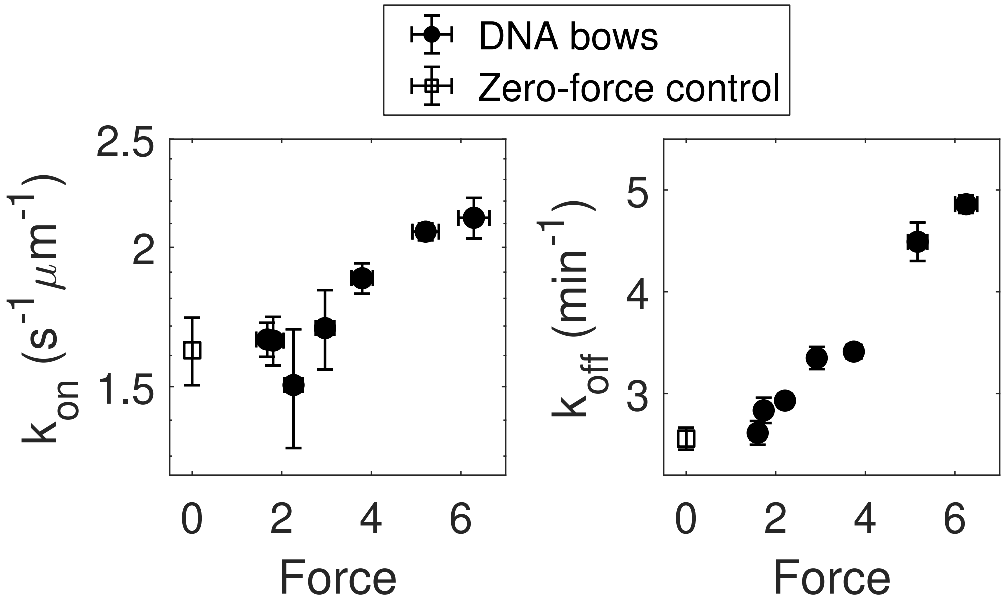

Supplementary Figure S10. Comparison of mean unbinding rates using DNA bows with a "zero-force" control molecule. Zero-force molecules were constructed by annealing Hairpin 2 with the Reverse Cy3 primer used in our bow experiments. Closed circles depict DNA bow measurements, while the open square depicts linear construct measurements. For all points, the error bars display the standard error of the mean. Three independent trials were performed for all bow sizes as well as for the control molecule.

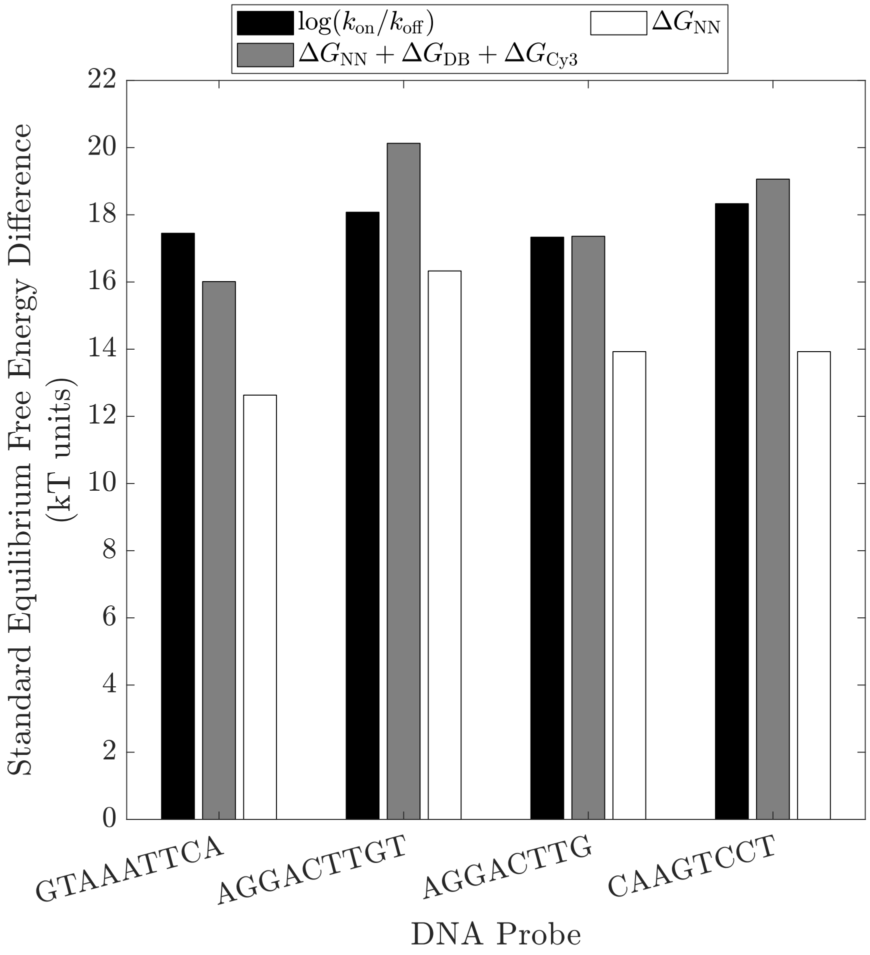

Supplementary Figure S11. A comparison of the measured standard equilibrium free energy difference values of all DNA probes to their corresponding nearest-neighbor predictions. The free energy difference, $\Delta G = \log(k_{on}/k_{off})$, was calculated using the average k_{off} and k_{on} values observed for the 252 bp DNA bow (black bars). The predicted free energy difference of a freely diffusing 8 bp to 9 bp duplex, ΔG_{NN}, was then calculated using published nearest-neighbor thermodynamic parameters (white bars) [85]. We then modified this estimate to correct for our experimental conditions by adding the energy contribution of dangling base stacking interactions ΔG_{DB} [70] as well as the energy contribution of a Cy3 dye attachment ΔG_{Cy3} (gray bars) [74]. Both estimates were calculated at a temperature 22 °C to match our experiment. The resulting sum was corrected for the monovalent cation concentration of our buffer ($[Mono^+] = [Na^+] + [Tris^+] = 150\,mM$) using the calibration formula published by SantaLucia Jr. Note that this estimate assumes that half of Tris molecules are protonated [86].

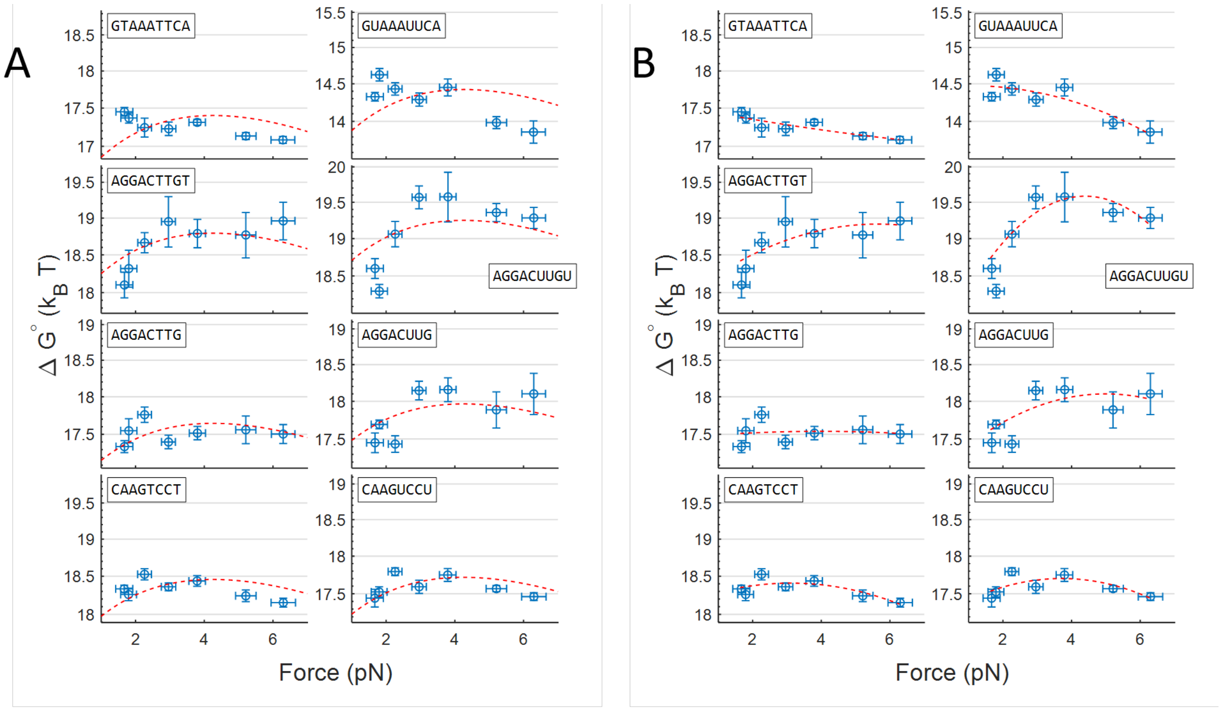

Supplementary Figure S12. Force dependence of the equilibrium free energy difference $\Delta G = \log(k_{\mathrm{on}}/k_{\mathrm{off}})$ between the bound and unbound states. Here, ΔG is defined as the additional free energy of the unbound state relative to the bound state. The force exerted by each bow was calculated with Equation 2, using the mean extension $\overline{x}$ of the bow's end-to-end distance distribution. Vertical error bars represent the standard error of the mean; horizontal error bars were calculated using $\left.\frac{\partial f(x)}{\partial x}\right|_{\overline{x}} \cdot \sigma(x)$, where $\overline{x}$ and $\sigma(x)$ are the mean and standard deviation of the bow's end-to-end distance distribution.

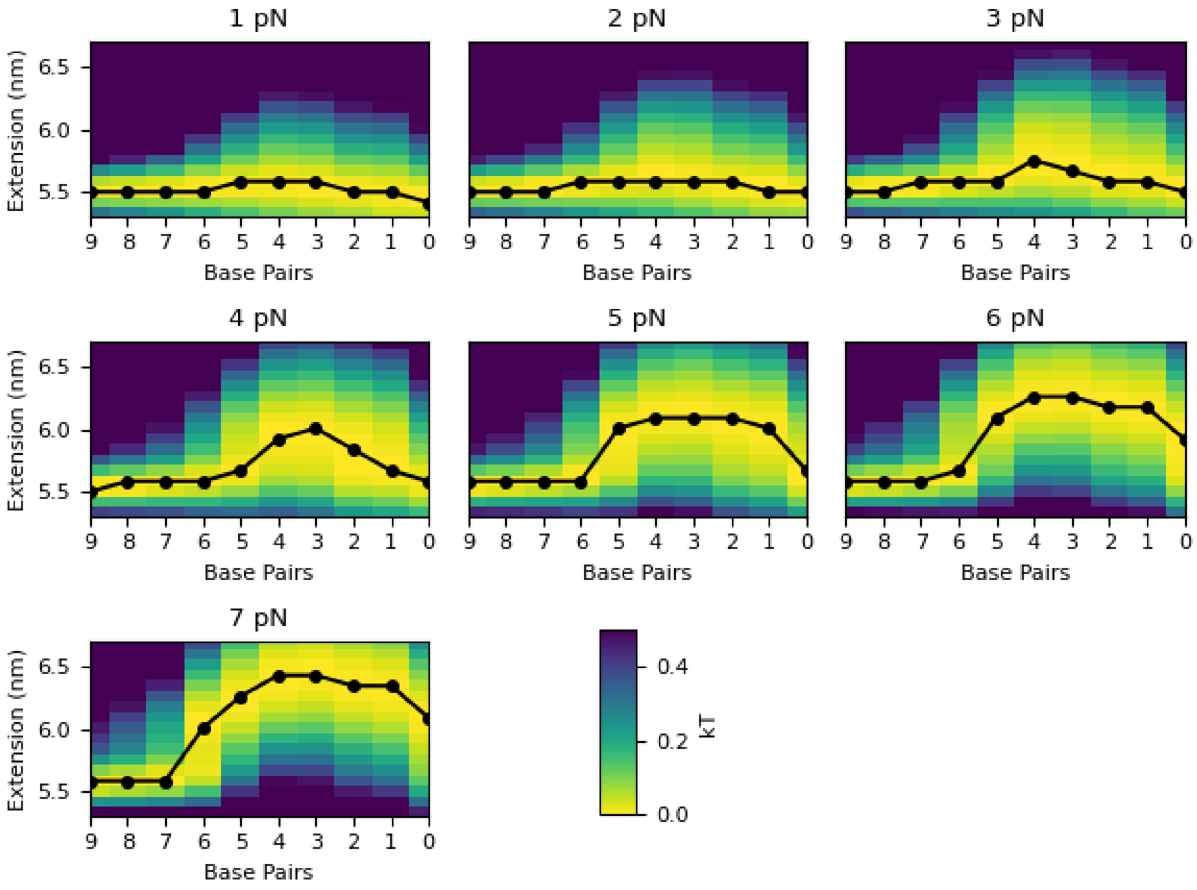

Supplementary Figure S13. Extension free energy difference heat map, calculated from VMMC simulations with umbrella sampling. Cells display the free energy difference along the extension coordinate relative to the minimum energy value of the given base pair column. Solid black dots indicate the extension location of the minimum free energy value.

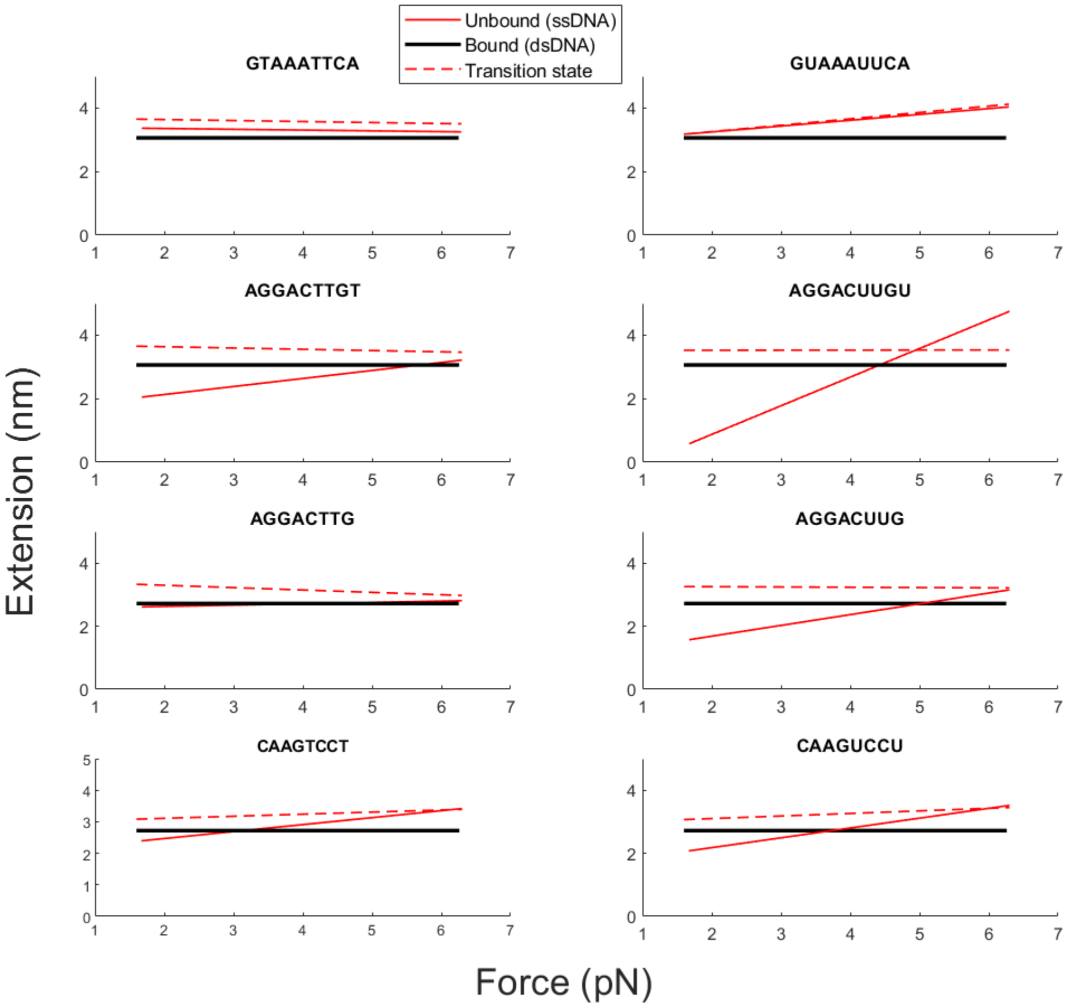

Supplementary Figure S14. Force-extension relations (FER) produced by the fitting parameters in Supplementary Table S4. Negative slopes seen in some FERs, which are unphysical, should be interpreted as zero slope or extremely large κ.

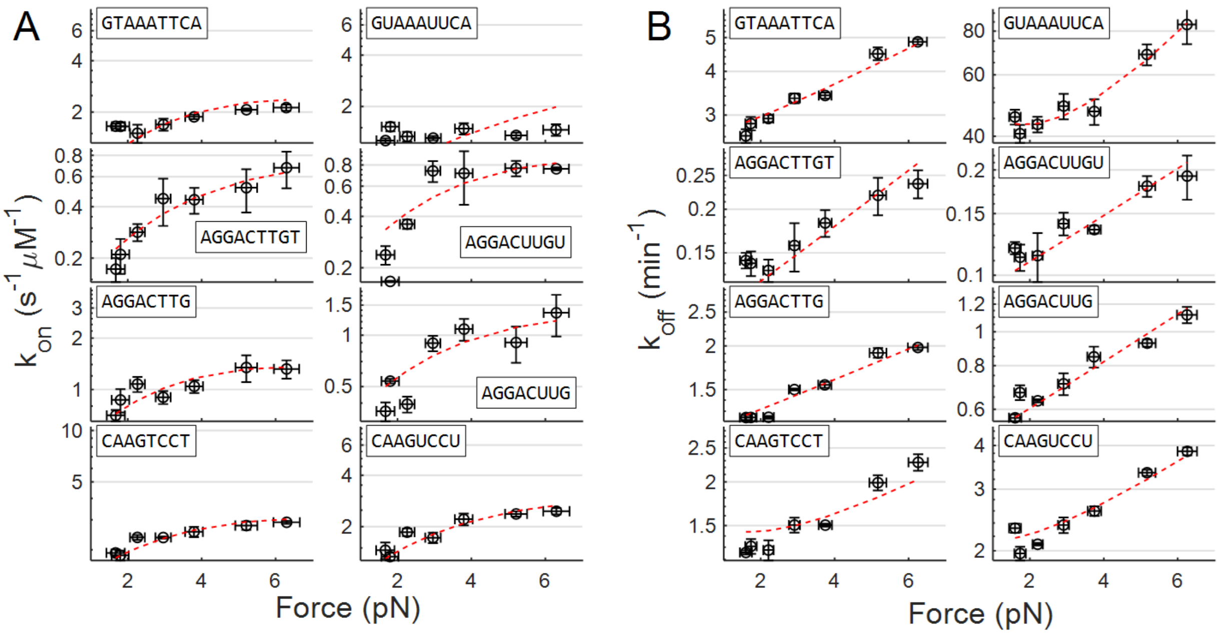

Supplementary Figure S15. Fitting of force-dependent rates using a model based on the Marko-Siggia formula. In this model, all three states are assumed to obey the force-extension curve given by the Marko-Siggia formula (Supplementary Equation S1). The persistence length (P) and the contour length per nucleotide (h) were fixed to 53 nm and 0.34 nm for the bound state, and 1.32 nm and 0.6 nm for the unbound state. The transition state parameters ($P_\ddagger$ and $h_\ddagger$) were allowed to vary for each sequence to fit the model simultaneously to the corresponding binding and unbinding rates. The two additional fitting parameters are $k_{\text{on}}(0)$ and $k_{\text{off}}(0)$, which set the offsets along the y-axis. The fitted curves are shown in red dashed lines, and the parameters obtained from the fitting are listed in the Supplementary Table S5.

DNA bow arc duplex segments ($5'$ to $3'$)	
74 bp	GACTCCCCACTCGTCGTACGAGGTCGCACACGCCCCACACCCAGACCTCCCTGCCCTGGTACCTC AGCACTGAG
84 bp	GACTCCCCACTCGTCGTACGCAACGAGGTCGCACACGCCCCACACCCAGACCTCCCTGCGAGCGC CTGGTACCTCAGCACTGAG
105 bp	GACTCCCCACTCGTCGTACGATCGCCATGGCAACGAGGTCGCACACGCCCCACACCCAGACCTCC CTGCGAGCGGGCATGGGTACCCTGGTACCTCAGCACTGAG
126 bp	GACTCCCCACTCGTCGTACCACCCACGCGCGATCGCCATGGCAACGAGGTCGCACACGCCCCACA CCCAGACCTCCCTGCGAGCGGGCATGGGTACAATGTCCCCGCCTGGTACCTCAGCACTGAG
158 bp	GACTCCCCACTCGTCGTACGTTTGGGGAAAGACCACACCCACGCGCGATCGCCATGGCAACGAGG TCGCACACGCCCCACACCCAGACCTCCCTGCGAGCGGGCATGGGTACAATGTCCCCGTTGCCACA GAGACCACCCTGGTACCTCAGCACTGAG
210 bp	GACTCCCCACTCGTCGTACTGCGAAATCCGGAGCAACGGGCAACCGTTTGGGGAAAGACCACACC CACGCGCGATCGCCATGGCAACGAGGTCGCACACGCCCCACACCCAGACCTCCCTGCGAGCGGGC ATGGGTACAATGTCCCCGTTGCCACAGAGACCACTTCGTAGCACAGCGCAGAGCGTAGCGCCTGG TACCTCAGCACTGAG
252 bp	GACTCCCCACTCGTCGTACTTTTTGTTTACGCGACAACTATGCGAAATCCGGAGCAACGGGCAAC CGTTTGGGGAAAGACCACACCCACGCGCGATCGCCATGGCAACGAGGTCGCACACGCCCCACACC CAGACCTCCCTGCGAGCGGGCATGGGTACAATGTCCCCGTTGCCACAGAGACCACTTCGTAGCAC AGCGCAGAGCGTAGCGTGTTGTTGCTGCTGACAAAAGCCTGGTACCTCAGCACTGAG
**Primers for making DNA force assay duplex segments ($5'$ to $3'$)**	
	↓ 20 nt
74 Forward	GACTCCCCACTCGTCGTACGAGGTCGCACACGCC
84 Forward	GACTCCCCACTCGTCGTACGCAACGAGGTCGCACAC
105 Forward	GACTCCCCACTCGTCGTACGATCGCCATGGCAACG
126 Forward	GACTCCCCACTCGTCGTACCACCCACGCGCGAT
158 Forward	GACTCCCCACTCGTCGTACGTTTGGGGAAAGACCACAC
210 Forward	GACTCCCCACTCGTCGTACTGCGAAATCCGGAGCA
252 Forward	GACTCCCCACTCGTCGTACTTTTTGTTTACGCGACAACTATG
74 Reverse	CTCAGTGCTGAGGTACCAGGGCAGGGAGGTCTGGGTG
84 Reverse	CTCAGTGCTGAGGTACCAGGCGCTCGCAGGGAGGT
105 Reverse	CTCAGTGCTGAGGTACCAGGGTACCCATGCCCGCTC
126 Reverse	CTCAGTGCTGAGGTACCAGGCGGGGACATTGTACCCATG
158 Reverse	CTCAGTGCTGAGGTACCAGGGTGGTCTCTGTGGCAACG
210 Reverse	CTCAGTGCTGAGGTACCAGGCGCTACGCTCTGCGCT
252 Reverse	CTCAGTGCTGAGGTACCAGGCTTTTGTCAGCAGCAACAACA
**Primers for making circular molecules, target segment underlined ($5'$ to $3'$)**	
T1	[Phos]TTTTGAATTTACTTTGACTCCCCAC[BiotindT]CGTCGTAC
T2 & T3	[Phos]TTTACAAGTCCTTTTGACTCCCCAC[BiotindT]CGTCGTAC

T4	[Phos]TTTAGGACTTGTTTTGACTCCCCAC[BiotindT]CGTCGTAC
Reverse	[Phos]CTCAGTGC|TGAGGTACCAGG
**Primers for making Cy3-labeled molecules for strand exchange (5′ to 3′)**	
Forward	GACTCCCCACTCGTCGTAC
Reverse (Cy3)	[Cy3]CTCAGTGCTGAGGTACCAGG
**DNA hairpin control molecules**	
Hairpin 1	[BIOTEG]CCTGGTACCTCAGCACTGAGTTTACAAGTCCTTTTGACTCCCACCGTCGTTTTCGACGGTGGGAGTC
Hairpin 2	[BIOTEG]CCTGGTACCTCAGCACTGAGTTTTGAATTTACTTTGACTCCCACCGTCGTTTTCGACGGTGGGAGTC
Cy5 acceptor probes (5′ to 3′)	
**DNA and RNA probes for smFRET experiments (5′ to 3′)**	
P1-DNA	[Cy5]GTAAATTCA
P1-RNA	[Cy5]GUAAAUUCA
P2-DNA	[Cy5]AGGACTTGT
P2-RNA	[Cy5]AGGACUUGU
P3-DNA	[Cy5]AGGACTTG
P3-RNA	[Cy5]AGGACUUG
P4-DNA	[Cy5]CAAGTCCT
P4-RNA	[Cy5]CAAGUCCU

Supplementary Table S1: List of DNA sequences, PCR primers, and DNA/RNA probes. All bow arc duplex segments are sourced from yeast genomic DNA, and extended to include common adapter sequences on each end. Forward primers for making circular DNA include the 15 nt sequence containing the 9 nt ssDNA complementary target segment (underlined); the reverse primer for making circular DNA includes the nick site (marked with a vertical line "|"). DNA and RNA probes were added to imaging buffer at 20 nM during smFRET experiments to measure unbinding (k_{off}) and binding rates (k_{on}).

End-to-end extension (nm), $\overline{x} \pm \sigma(x)$							
Unbound state							
Target	74 bp	84 bp	105 bp	126 bp	158 bp	210 bp	252 bp
1	5.7 ± 0.9	5.5 ± 0.9	5.3 ± 0.9	5.3 ± 0.9	5.2 ± 0.9	5.2 ± 0.9	5.2 ± 0.9
2 & 3	5.6 ± 0.9	5.5 ± 0.9	5.3 ± 0.9	5.3 ± 0.9	5.2 ± 0.9	5.2 ± 0.9	5.2 ± 0.9
4	5.6 ± 1.0	5.5 ± 0.9	5.3 ± 0.9	5.3 ± 0.9	5.2 ± 0.9	5.2 ± 0.9	5.2 ± 0.9
Bound state							
Target	74 bp	84 bp	105 bp	126 bp	158 bp	210 bp	252 bp
1	5.8 ± 0.7	5.7 ± 0.7	5.5 ± 0.7	5.5 ± 0.7	5.5 ± 0.6	5.5 ± 0.6	5.5 ± 0.6
2	5.7 ± 0.7	5.6 ± 0.7	5.5 ± 0.7	5.5 ± 0.7	5.5 ± 0.7	5.5 ± 0.6	5.5 ± 0.6
3	5.8 ± 0.7	5.6 ± 0.7	5.6 ± 0.7	5.5 ± 0.7	5.4 ± 0.7	5.5 ± 0.7	5.5 ± 0.7
4	5.7 ± 0.7	5.6 ± 0.7	5.5 ± 0.7	5.5 ± 0.7	5.4 ± 0.7	5.4 ± 0.6	5.5 ± 0.6

Supplementary Table S2. Mean ($\overline{x}$) and standard deviation values ($\sigma(x)$) of the end-to-end distance x of each DNA bow in both the probe-bound and probe-unbound state. Values were calculated using the measured x distance values of 7.5×10^4 configurations saved over $t = 1.14\,\mu s$ of simulation time. x is defined as the distance between backbone sites on the terminal bases of the elastic arc that are covalently linked to the ssDNA target segment.

**Order Parameter**	**Weight**
0 bp	221.516
1 bp	1107580
2 bp	193328
3 bp	45596.1
4 bp	8774.79
5 bp	1459.08
6 bp	206.943
7 bp	32.1335
8 bp	2.65784
9 bp	1

Supplementary Table S3. Umbrella sampling weight values for Monte Carlo DNA melting simulations. The potential of the system for all order parameter values of melting shown in the left column above were biased with unique weight values, as shown in the right column. Afterward, the unbiased probabilities were calculated using 6.

	$1/a_\ddagger$ ($nm \cdot pN^{-1}$)	$b_\ddagger$ (nm)	$1/a_u$ ($nm \cdot pN^{-1}$)	b_u (nm)	$k_{on}(0)$ ($\mu M^{-1} \cdot s^{-1}$)	$k_{off}(0)$ (s^{-1})
GTAAATTCA	-0.032	3.70	-0.025	3.41	1.40	2.10
AGGACTTGT	-0.040	3.72	0.252	1.63	0.10	0.10
AGGACTTG	-0.075	3.45	0.042	2.54	0.58	0.93
CAAGTCCT	0.066	2.98	0.221	2.03	1.38	1.11
GUAAAUUCA	0.201	2.86	0.183	2.88	1.38	43.59
AGGACUUGU	0.002	3.52	0.901	-0.93	0.06	0.09
AGGACUUG	-0.009	3.27	0.342	1.00	0.20	0.49
CAAGUCCU	0.081	2.94	0.312	1.56	0.87	1.86

Supplementary Table S4. Fitting parameters of the force-dependent rates using a linear model (Equation 10). The parameters a_α and b_α correspond to the stiffness constant and relaxed extension of the unbound ($\alpha = u$), or transition state ($\alpha = \ddagger$). $k_{on}(0)$ and $k_{off}(0)$ are the zero-force rates for binding and unbinding, respectively.

	$P_\ddagger$ (nm)	$h_\ddagger$ (nm)	$k_{on}(0)$ ($\mu M^{-1} \cdot s^{-1}$)	$k_{off}(0)$ (s^{-1})
GTAAATTCA	26.1	0.41	0.60	2.7
AGGACTTGT	53	0.43	0.09	0.1
AGGACTTG	51.6	0.39	0.35	1.1
CAAGTCCT	6.5	0.45	1.23	1.8
GUAAAUUCA	3.3	0.56	0.62	61.4
AGGACUUGU	53	0.41	0.14	0.1
AGGACUUG	53	0.42	0.22	0.5
CAAGUCCU	8.6	0.46	0.81	2.4

Supplementary Table S5. Fitting parameters of the force-dependent rates using a model based on the Marko-Siggia formula (Equation S1). $P_\ddagger$ and $h_\ddagger$ correspond to the persistence length and contour length per nucleotide of the molecule in its transition state. $k_{on}(0)$ and $k_{off}(0)$ are the zero-force rates for binding and unbinding respectively.
